# Integrated inference and evaluation of host–fungi interaction networks

**DOI:** 10.3389/fmicb.2015.00764

**Published:** 2015-08-04

**Authors:** Christian W. Remmele, Christian H. Luther, Johannes Balkenhol, Thomas Dandekar, Tobias Müller, Marcus T. Dittrich

**Affiliations:** ^1^Department of Bioinformatics, University of WürzburgWürzburg, Germany; ^2^Department of Human Genetics, University of WürzburgWürzburg, Germany

**Keywords:** pathogen–host interaction (PHI), protein–protein interaction, interolog, *Candida albicans*, *Aspergillus fumigatus*, network inference, pathogenicity, bioinformatics and computational biology

## Abstract

Fungal microorganisms frequently lead to life-threatening infections. Within this group of pathogens, the commensal *Candida albicans* and the filamentous fungus *Aspergillus fumigatus* are by far the most important causes of invasive mycoses in Europe. A key capability for host invasion and immune response evasion are specific molecular interactions between the fungal pathogen and its human host. Experimentally validated knowledge about these crucial interactions is rare in literature and even specialized host–pathogen databases mainly focus on bacterial and viral interactions whereas information on fungi is still sparse. To establish large-scale host–fungi interaction networks on a systems biology scale, we develop an extended inference approach based on protein orthology and data on gene functions. Using human and yeast intraspecies networks as template, we derive a large network of pathogen–host interactions (PHI). Rigorous filtering and refinement steps based on cellular localization and pathogenicity information of predicted interactors yield a primary scaffold of fungi–human and fungi–mouse interaction networks. Specific enrichment of known pathogenicity-relevant genes indicates the biological relevance of the predicted PHI. A detailed inspection of functionally relevant subnetworks reveals novel host–fungal interaction candidates such as the *Candida* virulence factor PLB1 and the anti-fungal host protein APP. Our results demonstrate the applicability of interolog-based prediction methods for host–fungi interactions and underline the importance of filtering and refinement steps to attain biologically more relevant interactions. This integrated network framework can serve as a basis for future analyses of high-throughput host–fungi transcriptome and proteome data.

## Introduction

Fungal pathogens infect hundreds of millions of people world-wide every year (Havlickova et al., 2008). Although, the death toll of fungal diseases is comparable to that of malaria or tuberculosis the global burden imposed by fungal pathogens still remains underestimated (Brown et al., 2012). In general, infections caused by fungal pathogens can lead to a diverse range of diseases ranging from superficial infections to invasive mycoses. The outcome of fungal infections is often associated with the intactness of the patients’ immune system and therefore fungi pose an increasingly severe threat to the growing numbers of immunocompromised patients in modern medicine, with high mortality rates exceeding 50% for invasive fungal diseases (Brown et al., 2012).

Among fungal pathogens the dimorphic yeast *Candida albicans* and the filamentous fungus *Aspergillus fumigatus* are the most important causes of life-threatening invasive mycoses (Horn et al., 2012). *C. albicans* colonizes the skin and intestinal mucosa of 30–70% of healthy individuals and invasive infection almost exclusively begins endogenously starting from a usually harmless surface colonization, frequently in the gastrointestinal tract (Gow et al., 2012). In contrast to the endogenous pathway of *C. albicans*, infections by *A. fumigatus* mainly occur exogenously via the inhalation of fungal spores (conidia) causing chronic pulmonary aspergillosis or invasive aspergillosis in patients with a severely weakened immune system (Brown et al., 2012). Despite these differences during the infection process, several common strategies of pathogenesis are shared between both fungi.

Host–fungi interactions have been described as commensalism, symbiosis, or pathogenicity. Interestingly, the mechanisms of symbiosis and pathogenicity share common features and there is evidence for parallel trends in evolution between host and pathogens (Ochman and Moran, 2001). The transition from commensal to pathogen is often dependent on small differences (Martin and Nehls, 2009) and the host–pathogen relation can change by environmental conditions (Hube, 2004). Strong adhesion of the fungi to the surface forming a protective biofilm is important for invasive growth, as invasion is driven by pressure on the solid substrate (de Groot et al., 2013). In this sense host–fungal interaction can be characterized by the formation of symbiotic or pathogenic interfaces (Bonfante and Genre, 2010). This relates in particular to processes of pathogen–host interaction (PHI) where both fungi mainly need to overcome similar epithelial barriers and develop skills for the evasion of the innate immune system, capabilities which contribute to the aggressiveness of both pathogens (Horn et al., 2012).

Therefore, a principal aim of systems biological research of human–pathogenic fungi is to unravel the intricate network of interactions between host and the fungal pathogen and elucidate the complex pathogenesis processes of fungal infections. A major quest in this field is the identification of physical or direct interactions between fungus and host proteins during the infection processes. Albeit the research of host–pathogen interactions is becoming increasingly popular in experimental as well as computational science, only a small number of interactions between fungi and human have been reported in literature so far. This leaves a large gap for novel bioinformatical strategies for the prediction of PHI of pathogenic fungi.

With the advent of large scale interaction detection methods the experimental and computational analyses of protein–protein interactions (PPIs) have established an important research field in bioinformatics during the last decade. Still most efforts have been dedicated to the investigation of intraspecies interactions (i.e., interaction between proteins within one species). The primary species in the focus of investigation so far have been *Homo sapiens* and *Saccharomyces cerevisiae*. This is reflected in the fact that the largest experimentally derived PPI datasets available in databases primarily cover *H. sapiens* and *S. cerevisiae* interactions. Currently, these two species constitute almost 74% percent of all non-redundant physical interactions^1^ (*H. sapiens*: 50.7% and *S. cerevisiae*: 23.0%) in the BioGRID database (Chatr-Aryamontri et al., 2013). The networks of most other species are considerably smaller and for network analysis these datasets are often extended by the inclusion of interolog based predictions to obtain a larger search space, where interologs are defined as PPIs that are conserved between orthologous proteins in different species (Walhout et al., 2000). Nowadays the interolog approach is commonly used for the classical prediction intraspecies interactions and is particularly valuable for the prediction of novel PPI in species where only a small number of interactions have been experimentally detected. Conceptually, the interactions are transferred from one species to another. This means that if for a given pair of interacting proteins in the source species, homologues for both interaction partners exist in the target species an interaction between those two homologs is inferred. The rational of this interaction transfer is based on the assumption that if a pair of homologous proteins originates from the same ancestral pair of interacting proteins, it can be expected, that the inheritance of the amino acid sequence translates into a related and similar protein structure, and thereby the capability of mutual interaction is also inherited from the ancestral interacting proteins (Walhout et al., 2000). This approach has been extended to the prediction of interspecies interactions and in particular to the prediction of PHIs (Zhou et al., 2013a).

Recent studies investigated the interaction between *H. sapiens* and *Plasmodium falciparum* (Dyer et al., 2007; Lee et al., 2008; Wuchty, 2011), *H. sapiens* and *Helicobacter pylori* (Tyagi et al., 2009), *H. sapiens* and *E. coli* (Krishnadev and Srinivasan, 2011), *H. sapiens* and *Salmonella enterica* (Krishnadev and Srinivasan, 2011) and *H. sapiens* and *Yersinia pestis* (Krishnadev and Srinivasan, 2011) as well as between *H. sapiens* and *Mycobacterium tuberculosis* (Zhou et al., 2014). Apart from the more frequently investigated protozoan *P. falciparum*, most of these studies focus on the interaction with a bacterial pathogens. Fungal infections have only rarely been researched. A recent study examined the interaction between zebra fish and *Candida* (Chen et al., 2013), however, a systemic investigation of direct host–pathogen-PPI between the fungi either *C. albicans* or *A. fumigatus* and the human host has to our knowledge not be conducted so far.

Here we present an extended interolog-based method for the prediction of fungal–host interactions. We focus on the clinically most relevant fungi, the dimorphic yeast *C. albicans* and the filamentous fungus *A. fumigatus*. In addition to the human host, we also investigate interactions between these fungi and *Mus musculus*, since it is the most frequently used animal model in medical sciences. As basic interolog prediction approaches for cross-species analysis often produce large initial predictions sets, we develop and establish an advanced filtering and selection strategy, to reduce the initial set of raw predictions to a smaller refined set of high quality predictions. To this end, we integrate information on cellular localization of the predicted host and pathogen interaction partners and focus on proteins associated with cellular functions with relevance for the infection process. The enrichment of established infection and pathogenicity related genes during these subsequent refinement steps emphasizes the biological relevance of the predicted PHIs, from which we highlight and describe some promising candidate interaction in more detail. By this, we demonstrate the benefit of the interolog-based approach in combination with advanced filtering and refinement steps for prediction fungal-host interactions.

## Materials and Methods

### Template Intraspecies Interaction Networks

For the host–fungi interaction network inference, the intraspecies interaction data of *S. cerevisiae* and *H. sapiens* were downloaded from the following 14 active partners of the International Molecular Exchange (IMEx) consortium (Orchard et al., 2012):

DIP (Salwinski et al., 2004), IntAct (Orchard et al., 2014), MBInfo^2^, MINT (Licata et al., 2012), MatrixDB (Chautard et al., 2011), Molecular Connections^3^, I2D (Brown and Jurisica, 2007), InnateDB (Breuer et al., 2013), UCL-BHF group, UCL London^4^, UniProt Swiss-Prot group, SIB (The UniProt Consortium, 2014), BioGRID (Chatr-Aryamontri et al., 2013), MPact (Pagel et al., 2005), BIND (Bader et al., 2001), and MPIDB (Goll et al., 2008).

PSICQUIC queries (Aranda et al., 2011) were used to retrieve human and yeast intraspecies interaction information from this databases on 09/09/2014. Non-coding genes, interaction loops of self-interacting proteins as well as interactions of the interaction types “colocalization,” “additive genetic interaction defined by inequality,” “suppressive genetic interaction defined by inequality,” “synthetic genetic interaction defined by inequality,” “genetic interaction,” “genetic inequality,” “genetic interference,” and “self-interaction” were not used for the template networks.

### Orthology Information

Orthology information for *C. albicans*, *S. cerevisiae*, *H. sapiens*, *M. musculus*, and *A. fumigatus* was downloaded from InParanoid8 (Sonnhammer and Ostlund, 2014). Additionally, orthology relations between *A. fumigatus* and *S. cerevisiae* were retrieved from Aspergillus Genome Database (AspGD; Cerqueira et al., 2014). Orthologies of the species pair *A. fumigatus* and *H. sapiens* which was neither available from InParanoid8 nor AspGD, were computed via the InParanoid version 4.1^5^ using parameters comparable to the parameters of similar species pairs (*H. sapien*s – *A. kawachii*). Blast version 2.2.26 with the scoring matrix Blosum62, a score-cutoff of 40 bits, a sequence overlap of 0.5, a group merging cutoff 0.5 and a minimal score of 0.05 was used as InParanoid settings. The dataset for *A. fumigatus* protein sequence was downloaded from AspGD, while the protein sequences of *H. sapiens* originated from the InParanoid8 server.

### Gene Ontology

Gene Ontology (GO) slim annotations, a subset of the GO dataset (Ashburner et al., 2000) were used to categorize genes in host–fungi interactions of the inferred networks regarding three domains: biological process, molecular function and cellular component. GO slim associations were retrieved from the Candida Genome Database (CGD; Arnaud et al., 2005) and the AspGD (Cerqueira et al., 2014) for both fungal pathogen species. GO slim associations for the host species (*H. sapiens* and *M. musculus*) were downloaded from EnsEmbl 76 (Flicek et al., 2014).

Genes of the inferred fungi–host interaction networks were categorized by GO slim cellular component annotation in likely and unlikely host–fungal interactors under the assumption that interacting host and fungal proteins have to be localized on potential interface (e.g., cell surface or endosome membrane). The GO slim cellular component terms for likely interspecies interactions on the fungal and host side were listed in **Table 1**.

**Table 1:** Numbers of genes in the primary predicted host–fungal PPI networks belonging to the cellular component GO filter terms.

(A) Filter terms for host side		

**GO slim cellular component terms**	**Number of genes in *Homo sapiens***	**Number of genes in *Mus musculus***
Extracellular region	2,566	783
Plasma membrane	2,310	2,024
Extracellular space	631	419
Endosome	476	421
Lysosome	306	247
Cilium	138	202
Proteinaceous extracellular matrix	115	132
External encapsulating structure	3	5
Only other GO terms	6,531	7,645
No GO terms	902	361

**(B) Filter terms for fungi side**		

**GO slim cellular component terms**	**Number of genes in *Aspergillus fumigatus***	**Number of genes in *Candida albicans***

Plasma membrane	270	236
Extracellular region	94	33
Cell wall	52	74
Only other GO terms	3214	3160
No GO terms	0	1114

**(C) Sizes of host–fungi PPI networks after localization refinement**				

**Host species**	**Pathogen species**	**Number of host–pathogen interactions**	**Number of host interactors**	**Number of pathogen interactors**	

*H. sapiens*	*A. fumigatus*	17,853 (8.4%)	363 (10.2%)	2,393 (21.2%)	
*H. sapiens*	*C. albicans*	15,330 (4.3%)	301 (6.6%)	2,123 (19.2%)	
*M. musculus*	*A. fumigatus*	9,284 (4.5%)	337 (9.4%)	1,572 (14.9%)	
*M. musculus*	*C. albicans*	8,055 (2.4%)	282 (6.2%)	1,376 (13.3%)	

Similar to the refinement step for protein localization, proteins with pathogenicity-associated GO slim biological process terms were selected to enrich for pathogenicity-relevant interaction predictions (see **Table 2**). Only genes assigned to one of the referenced cellular component and biological process GO terms were used for further analyses.

**Table 2:** Numbers of genes in the primary predicted host–fungal PPI networks belonging to the biological process GO filter terms.

(A) Filter terms for host side		

**GO slim biological process terms**	**Number of genes in *H. sapiens***	**Number of genes in *M. musculus***
Signal transduction	951	602
Immune system process	491	255
Symbiosis, encompassing mutualism through parasitism	260	0
Cell adhesion	151	127
Circulatory system process	50	53
Only Other Slim BP annotations	1,246	878
No GOSlim BP annotation	0	0

**(B) Filter terms for fungi side**		

**GO slim biological process terms**	**Number of genes in *A. fumigatus***	**Number of genes in *C. albicans***

Pathogenesis	30	33
Cell adhesion	10	24
Biofilm formation	0	32
Interspecies interaction between organisms	0	30
Growth of unicellular organism as a thread of attached cells	0	2
Only Other Slim BP annotations	330	244
No GOSlim BP annotation	0	0

**(C) Sizes of host-fungi networks after functional refinement**					

**Host species**	**Pathogen species**	**Number of host–pathogen interactions**	**Number of host interactors**	**Number of pathogen interactors**

*H. sapiens*	*A. fumigatus*	1,137 (6.4%)	607 (25.4%)	33 (9.1%)	
*H. sapiens*	*C. albicans*	3,025 (19.7%)	840 (39.6%)	57 (18.9%)	
*M. musculus*	*A. fumigatus*	590 (6.4%)	355 (22.6%)	26 (7.7%)	
*M. musculus*	*C. albicans*	1,462 (18.2%)	461 (33.5%)	41 (14.5%)	

### Gene Ontology and Uniprot Tissue Enrichment

Interactors of subnetworks were tested for enriched GO annotation level 2 terms of the domains “biological process,” “cellular component,” “molecular function” (Ashburner et al., 2000) versus the GO terms background frequencies of the interactors in the full network. The functional enrichment tests were performed via the DAVID Bioinformatics Resources 6.7 (Huang da et al., 2009a,b) using GO terms of all levels and only reporting groups of the size of least two genes and an EASE Score Threshold (for gene-enrichment analysis modified Fisher Exact P-Value) of less than 0.1. The *p*-values were adjusted for multiple testing (Hochberg and Benjamini, 1990). Similar to the GO enrichment, the tissue enrichment analyses were performed on Uniprot tissue terms via the DAVID Bioinformatics Resources 6.7.

### Catalog of Pathogenicity-Relevant Genes

To get a set of genes of *H. sapiens* and *M. musculus* that are known to be involved in host–pathogen interactions, the PPI information were downloaded from the HPIDB version 5/22/2014 and the PATRIC database version Mar2013. Further, all interspecies interactions that involved viral pathogens or the interaction types which are not related to a direct PPI such as annotated as “colocalization,” “additive genetic interaction defined by inequality,” “suppressive genetic interaction defined by inequality,” “synthetic genetic interaction defined by inequality,” “genetic interaction,” or “genetic inequality” were removed from the dataset.

Also, the Victors database of PHIDIAS (Xiang et al., 2007), a database containing virulence factors originating from literature curation and bioinformatics analyses and the PHI-base (Winnenburg et al., 2008), a database containing expertly curated molecular and biological information on pathogenic genes experimentally verified to have an effect on the virulence outcome were searched for genes of the fungal pathogens *A. fumigatus* and *C. albicans* that are known as pathogenesis associated.

Additionally the public available interaction databases mentha (Calderone et al., 2013), HPIDB (Kumar and Nanduri, 2010), APID (Prieto and De Las Rivas, 2006), PHISTO (Durmus Tekir et al., 2013), PRIMOS (Rid et al., 2013), and the databases of IMEx (Orchard et al., 2012) were scanned to receive all already known interspecies interactions for human–*Candida*, human–*Aspergillus*, mouse–*Candida*, and mouse–*Aspergillus*.

To find already known human–*Aspergillus*, mouse–*Aspergillus*, human–*Candida*, and mouse–*Candida* interactions the public available interaction databases mentha, HPIDB, APID, PHISTO, PRIMOS, and the databases of IMEx was searched.

### Analysis of Dual RNA-Seq Data

For the comparison of predicted fungal–host interaction networks, gene expression data of a previously published time course of murine bone marrow derived dendritic cells phagocytosing *C. albicans* SC5314 cells was used (Tierney et al., 2012). The gene expression data constitutes of dual RNA-seq data simultaneously measuring the transcripts of *Candida* and mouse cells at 30, 60, 90, and 120 min post-infection. The sequenced reads were downloaded from http://www.ebi.ac.uk/arrayexpress/experiments/E-MTAB-595/. Contamination of poly-T at the read start and poly-A at the read end was removed via cutadapt version 1.6 (Martin, 2011). The curated reads were mapped on a combined reference of the *C. albicans* SC5314 version A22 (Arnaud et al., 2005) and the *M. musculus* version GRCm38.75 (Flicek et al., 2014) genome, using the short read mapping tool STAR version 2.4 (Dobin et al., 2013). For each gene of the *C. albicans* and the *M. musculus*, the uniquely mapped reads were counted with featureCounts version 1.4.3 (Liao et al., 2014). Fungal and host genes were tested for differential expression in the infection time course with DESeq2 version 1.6.2 (Love et al., 2014). Genes were identified as differentially expressed when they showed a significant (*p*-value <0.05) change in read counts after multiple testing correction (Hochberg and Benjamini, 1990).

### Network Visualization

The networks were visualized by Cytoscape (Shannon et al., 2003). The top 10% of fungal high degree interactors were removed from the visualized networks to improve the readability. The GO slim interaction network was based on grouping genes in GO slim groups that are annotated by the respective GO slim biological process terms. Improved readability of GO slim networks was achieved by merging GO slim groups fully contained in larger groups. Node size represents the number of genes contained in each GO slim term. Edge width and color depict number of interactions between two GO slim terms.

## Results

### Host–Fungi Interaction Data in Literature and Public Databases is Sparse

The primary objective of our work is to establish a comprehensive catalog of host–fungal interactions. A first literature search revealed that overall not much detailed data concerning PHIs for fungi is available so far. However, as PHIs have become an important topic in the last years, several databases for PHIs have been established. Up to date most of the interactions deposited in these databases still relate to viral and bacterial pathogens and almost no information concerning fungi is available at all. For example, the current HPIDB (Kumar and Nanduri, 2010) covers predominantly viral (74%: 29,942) and bacterial (22%: 8,992) pathogens and only 4% (1,628) of the interactions involve fungal species out of which over 92% (1,499) relate to *Saccharomyces* spp. To obtain a comprehensive overview of all host–fungi interaction data available so far, we first searched the content of the most prominent host–pathogen interaction databases [HPIDB, PHISTO (Durmus Tekir et al., 2013), and PRIMOS (Rid et al., 2013)] for established host–fungal interactions between human–*Candida*, human–*Aspergillus*, mouse–*Candida*, and mouse–*Aspergillus*. Nevertheless, the search returned only two distinct interactions between *C. albicans* and *H. sapiens* and one more for mouse–*Candida*: (i) *Candida* ORC1 (Origin recognition complex subunit 1) and human CDC23 (Cell division cycle protein 23), (ii) *Candida* Q00308 and human CD2BP2 (CD2 antigen cytoplasmic tail-binding protein 2). For the interaction between mouse and *Candida* only one interaction between the *Candida* CDC28 (Cyclin-dependent kinase 1) and murine Cdkn1b (Cyclin-dependent kinase inhibitor 1B) could be found. We could not find any interspecies interaction between human and *A. fumigatus* or between mouse and *A. fumigatus* from the above host–pathogen-databases. Therefore, we subsequently scanned APID (Prieto and De Las Rivas, 2006), mentha (Calderone et al., 2013) and all the 14 curated PPI databases of the IMEx consortium (Orchard et al., 2012) for cross-species interactions involving *A. fumigatus* and *C. albicans* (see Catalog of Pathogenicity-Relevant Genes section). This extended search revealed only one additional interspecies interaction that was not included in the PHI databases: *Candida* CDC42 (Cell division control protein 42 homolog) and the murine Scd2 (Acyl-CoA desaturase 2). No interactions for *A. fumigatus* have been found in above databases for human or mouse.

Since information in databases about PPIs between the fungal pathogens *C. albicans* and *A. fumigatus* and their hosts is sparse, we propose a framework to infer PHIs and thus create hypotheses for experimental validation.

### Dual Template Interolog-Based Host–Fungi PPI Network Inference Approach

**FIGURE 1:**
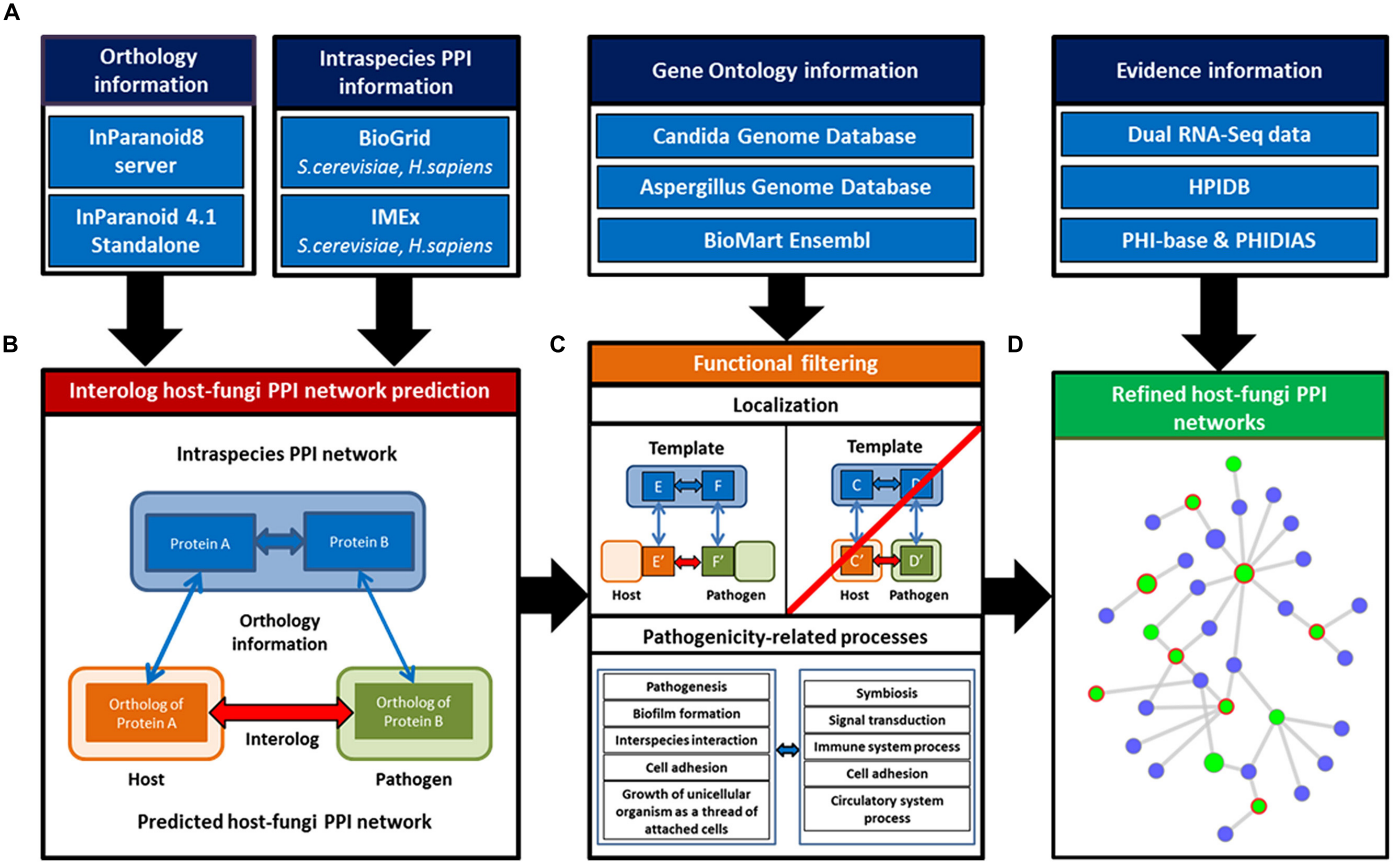
**Basic concept of the host–fungi PPI inference and refinement steps. (A)** Information of direct PPI from multiple public databases were integrated for the two template networks *Homo sapiens* and *Saccharomyces cerevisiae*. **(B)** These combined with orthology information allowed to identify host–fungi interologs. **(C)** Primary inferred networks were filtered for interactions which showed protein localizations pointing to possible interfaces between host and fungi. Additionally, the networks were refined for pathogenicity-related processes. **(D)** Evidence information of several independent sources (e.g., transcriptome data) were exploited to evaluate the refined host–fungi PPI networks.

The general approach applied in this study aims on the identification of novel potential PPIs between the selected host species *H sapiens* and *M. musculus* and the fungal pathogen species *C. albicans* and *A. fumigatus.* To derive these PHIs, we established an interolog-based inference method exploiting known intraspecies interactions in *H. sapiens* and *S. cerevisiae* as template networks combined with gene homology information between the template species and the host as well as the fungal species. Our approach comprises three steps which involve (i) the establishment of a comprehensive dual-species PPI template network, (ii) homology based inference of PHIs, and (iii) the application of an extended filtering strategy on the raw predictions to attain a core set of refined interaction predictions (see **Figure 1**).

#### Comprehensive Dual-Species PPI Template Network

To establish a comprehensive intraspecies template network for interspecies PHI interaction prediction we screened the BioGRID database (Chatr-Aryamontri et al., 2013) and 13 PPI databases associated with the Imex consortium for intraspecies PPIs in *H. sapiens* and *S. cerevisiae* resulting in 170,774 human interactions with 15,509 interactors and 272,167 yeast interactions with 5,824 interactors. As we primarily focus in this study on direct PPIs, the template networks were curated from PPIs detected by methods which are rather based on functional associations (e.g., “genetic interference”). Furthermore, all self-interactions were removed from this network. The resulting human and yeast intraspecies PPI networks consisted of 147,760 human interactions with 15,240 interactors and 130,665 yeast interactions with 5,789 interactors. Although the numbers of human interactions were reduced by almost 14%, the number of interactors barely decreased (1.7%). Since a large number of yeast interaction were identified by functional association methods, the number of interaction decreased by almost 52%, while similar to the human network the number of interactors was just reduced by less than 1% (see Supplementary Table S2).

#### Interolog-Based Prediction Yields Large Host Fungal Interaction Networks

Host–fungal interactions for each host–fungi pair were predicted based on the two template interaction networks. Thus, in a second step, we integrated the template interaction data with orthology information of the host, pathogen, and template species. Orthology information between the two template PPI networks of *H. sapiens* and *S. cerevisiae* and the host species *H. sapiens* and *M. musculus* as well as the fungal pathogens *C. albicans* and *A. fumigatus* was downloaded from the InParanoid 8 database (Sonnhammer and Ostlund, 2014), the species-specific genome databases (Binkley et al., 2014; Cerqueira et al., 2014; Costanzo et al., 2014) and missing species pairs complemented by orthology identification by the stand-alone program InParanoid 4.1 (Ostlund et al., 2010). For *H. sapiens* as template species, 16,582 mouse genes were identified as orthologs to 16,417 human genes, while 2,687 *Candida* genes were orthologs to 3,770 *H. sapiens* genes (2,808 *Aspergillus* and 4,277 *H. sapiens* genes, respectively). Interestingly, we found more than twice the number of *Candida* proteins being orthologs to yeast than orthologous *A. fumigatus* proteins, while the number between both fungi and human was comparable to *S. cerevisiae* – *A. fumigatus* orthologs (see Supplementary Table S1)

We searched for orthologs for both interactors of each template interaction to predict potential direct PPIs between the host species *H. sapiens* or *M. musculus* with the fungal pathogen species *C. albicans* or *A. fumigatus*. Interologs are PPIs inferred from one species to another by using orthology information (Walhout et al., 2000). In our approach, we simultaneously identified orthologs of one interactor in the host species and one interactor in the fungal species for each template interaction. The resulting cross-species interologs between the hosts and the pathogens should consequently have the potential to perform a PPI, given both interactors share the same location at one point in time. For the human-*Aspergillus* infection 213,518 interologs with 11,279 human and 3,576 *Aspergillus* interactors could be superimposed. Similar results were obtained for the three other infection setups human–*Candida*, mouse–*Aspergillus*, and mouse–*Candida* (see Supplementary Table S2).

#### Improving Primary Inferred Host–Fungi PPI Networks

Potential false predictions were reduced via refinement of the primary inferred host–fungi PPI networks based on functional data. Therefore, GO slim annotations of the cellular component and biological process (The Gene Ontology, 2014) were exploited in this filtering step. To enrich for likely interactions, only host and pathogen interactors which showed GO slim cellular component annotations pointing at locations associated to the cell surface and intracellular compartments which can be in direct host–fungi contact, were selected for the refined host–fungi PPI networks. The GO slim cellular compartment terms which were selected for filtering interactors based on their localization were summarized for the hosts (see **Table 1A**) and the fungi (see **Table 1B**). Only 902 human and 361 mouse genes showed no GO slim cellular component annotation at all. On the fungal side, this was the case for 1114 *Candida*, but none of *Aspergillus* genes. Altogether, only very few genes were lost in this filtering step due to missing localization information. The distribution of filtered GO slim cellular component terms clearly shows that the “extracellular region” is less abundant in the murine compared to the human interactor set (783 and 2566), while the other terms are similarly present between mouse and human. Surprisingly, the term “extracellular region” also shows a strong difference in distribution on the fungal side (94 *Aspergillus* interactors and 33 *Candida* interactors).

This filtering step reduced the interolog networks, e.g., human–*Aspergillus* with 213,518 interologs to 17,853 interactions with 2,393 human and 363 *Aspergillus* interactors. For all four interolog networks, the refinement step reduced the number of interactions to less than 9%, while the host interactors were reduced to less than 11% and the fungal interactors to less than 22%, respectively (see **Table 1C**).

In concordance with the localization filtering, a functional refinement utilizing representative biological process terms was applied. To improve the quality of the predicted network and increase the fraction of PPIs potentially associated to pathogenicity-relevant processes, we selected five GO slim biological process terms for filtering the host interactors (see **Table 2A**) and five GO slim biological process terms on the pathogen side (see **Table 2B**). All genes of the hosts and fungal pathogens showed an annotation of GO slim biological process.

In the localization-refined PPI networks, GO slim biological process annotations were available for each host and fungi interactor. Nonetheless, the number of human interactors assigned to the selected GO slim biological process terms was higher than for mouse. Especially, the GO slim term “Symbiosis, encompassing mutualism through parasitism” yielded the strongest difference with a coverage of 260 human interactors and 0 mouse interactors. For the fungal pathogens, the results were similar with fewer *A. fumigatus* interactors than *C. albicans* interactors assigned to selected GO biological process terms.

This filtering step reduced the localization-refined networks, e.g., mouse–*Candida* 8055 interactions with 1,376 mouse and 282 *Candida* interactors to 1,462 interactions with 461 mouse and 41 *Candida* interactors. For all four host–fungi networks, the refinement step reduced the number of interactions to less than 20%, while the host interactors were reduced to less than 40% and the fungal interactors to less than 19%, respectively (see **Table 2C**).

### The Dual Template Approach Substantially Enhances the Prediction Space for Host Fungal Network Inference

**FIGURE 2:**
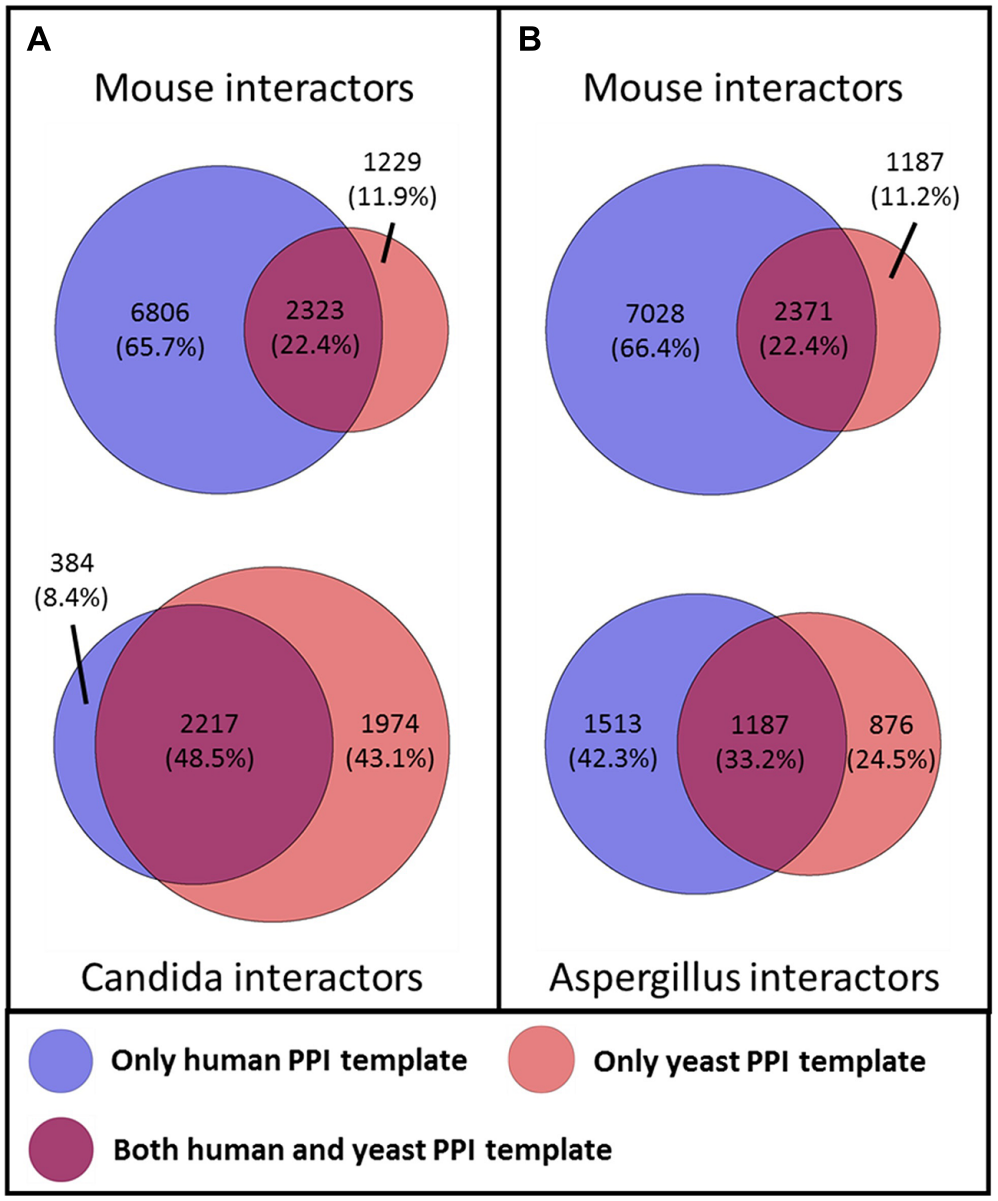
**Influence of the template networks on the predicted **(A)** mouse–*Candida* network **(B)** mouse–*Aspergillus* network.** The color of the circle denotes the template network from which the interactors originated.

To investigate the benefits of our dual-template approach for the interolog-based network inference, we examined for each host and fungal interactors the template network from which they were inferred. For this, we grouped the interactors of the primary inferred PHI networks based on their original template network (see **Figure 2**). On the host side, the human template exclusively makes up for 67.5% of the human interactors in the PHI networks, while over 10.2% of the human interactors originated only from the yeast template (see Supplementary Figure S1). About 22.3% of the human interactors were inferred from both the human and the yeast template. Similarly, for the mouse interactors, the human template solely makes up for over 66.0% of the murine interactors in the PHI networks, while more than 11.5% of the interactors originated only from the yeast template. About 22.4% of the murine interactors were inferred from both the human and the yeast template. Even though no orthology information was required for the inference of human interactors, we see similar distribution of template origin between human and murine interactors. On the fungal side, a substantially larger fraction of the *Aspergillus* interactors (24.4%) was inferred from yeast template, while the human template makes up for 42.4% of the *Aspergillus* interactors originating from the human template. Over 33.1% of the *Aspergillus* interactors were inferred from both the human and the yeast template. In contrast, only less than 8.5% of the *Candida* interactors were inferred from the human template, while more than 43.0% originated from yeast interologs. The largest fraction with more than 48.4% of the *Candida* interactors resulted from both human and yeast template. These numbers represent substantial differences in the distribution between both fungal pathogens, as could be expected by the smaller evolutionary distance from *S. cerevisiae to C. albicans* than from *S. cerevisiae* to *A. fumigatus*.

A GO enrichment analysis was performed for each group of interactors originating from human, yeast, or both template interaction networks compared to the whole set of interactors (see Supplementary Tables S3 and S4). The GO enrichment analyses showed that multiple GO categories related to PHI were significantly enriched in the human interactor subsets originating from the human template network (e.g., extracellular region part, cell adhesion, signal transducer activity) and yeast template network (e.g., membrane part, transmembrane transport, ion binding). Surprisingly, the subset of human interactors inferred by both template networks was enriched for GO categories of basic biological processes (e.g., intracellular part, ribonucleoprotein complex, nucleotide binding). Even with the overlap of subsets showing only few interesting enriched GO categories, the integration of both template networks complemented a large amount of significantly enriched pathogenicity-relevant categories (see Supplementary Table S3).

Similar to the host side, the GO enrichment analysis of the *Aspergillus* interactors predicted based on the human template network yielded significantly enriched pathogenicity-associated GO terms (e.g., oxidation reduction, ion binding). For the interactors originating from the yeast template network, a different set of pathogen-relevant GO terms (e.g., membrane, transferase activity) were enriched, while the *Aspergillus* interactors inferred by both template networks mainly basic biological processes were enriched (e.g., ribonucleoprotein complex, cellular metabolic process, structural constituent of ribosome; see Supplementary Table S4).

### Localization Filtering and Functional Refinement Improve Predicted Host–Fungi Networks

Since data on experimentally validated PHIs for fungal pathogens are rare and there is no golden standard for PHI network inference available, we created a dataset of pathogenicity-associated genes for validation of the refinement step. We extracted functional data encompassing (1) human and murine genes which have been reported to directly interact with pathogenic proteins (Kumar and Nanduri, 2010), (2) virulence and pathogenicity phenotypes induced by knock outs of fungal genes (Xiang et al., 2007; Winnenburg et al., 2008) and (3) infection responsive genes identified by analysis of a data set of an infection time course experiment of murine innate immune cells infected by *C. albicans* (Tierney et al., 2012).

#### Infection-Regulated Genes are Enriched in Resulting Host–Fungi Networks

**FIGURE 3:**
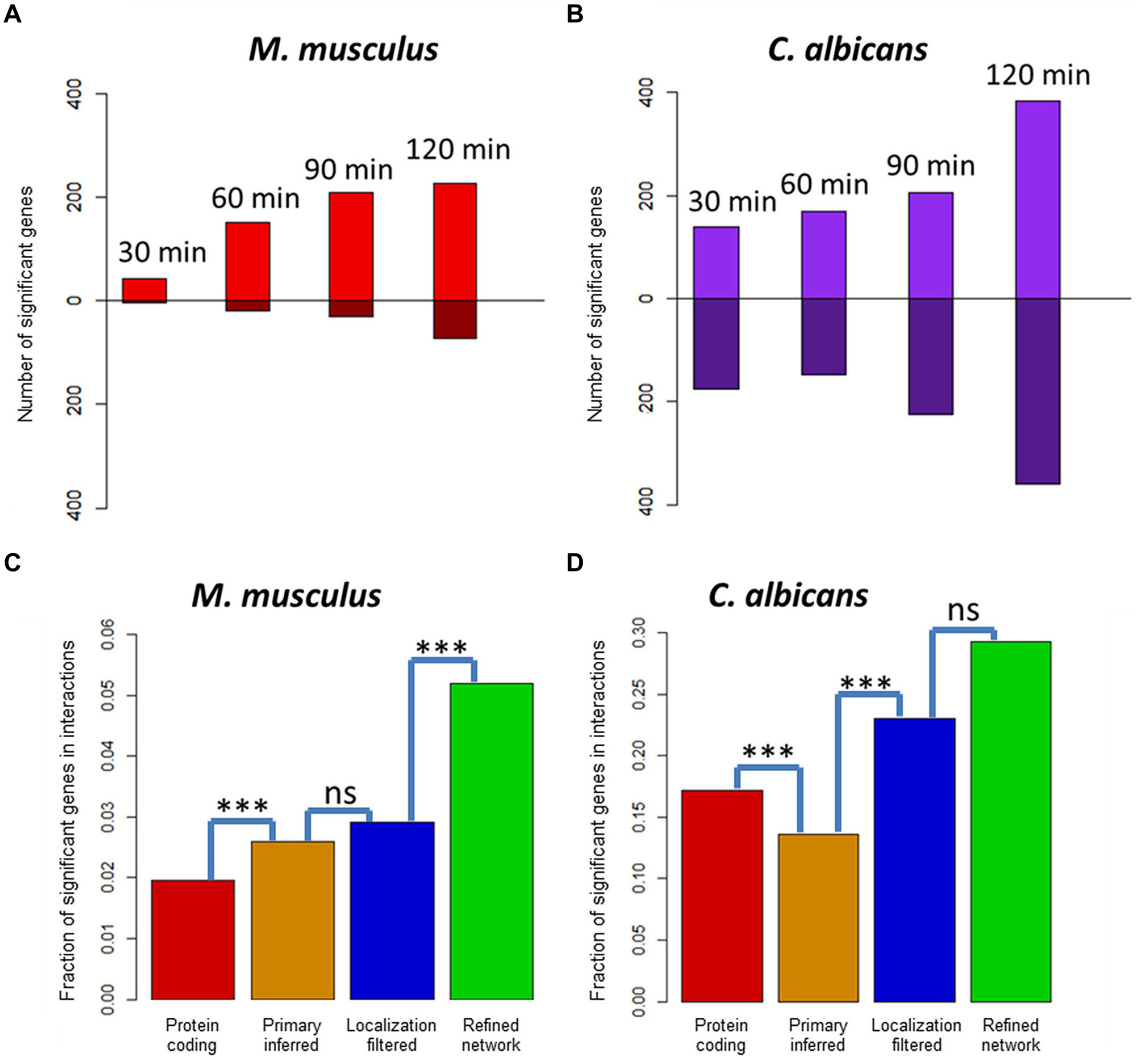
**Infection-regulated genes in predicted host and fungi interactors. (A)** Differentially expressed genes in murine innate immune cells 30, 60, 90, and 120 min post-infection with *Candida albicans* cells. Bars above the *x*-axis show the number of significantly upregulated genes, while bars below show the significantly downregulated genes. **(B)** Differentially expressed genes in *C. albicans* 30, 60, 90, and 120 min post-infection of murine innate immune cells. **(C)** Fraction of significantly deregulated genes in the sets of protein-coding genes, the primary inferred, the localization-filtered, and the functionally refined interactors of mouse. **(D)** Fraction of significantly deregulated genes in the sets of protein-coding genes, the primary inferred, the localization-filtered, and the biological process refined interactors of *C. albicans*. A test for enrichment of infection-regulated genes in the interactor sets after the primary inference, localization, and functional refinement step (Fisher exact test, ***p< 0.001).

Under the assumption, that deregulated genes over an infection time course are more likely to be involved in host–fungi interactions, exploiting transcriptomic or proteomic gene expression data can be used for the validation of the refinement step. The recently published simultaneous transcriptome sequencing of *C. albicans* and murine innate immune cells 0, 30, 60, 90, and 120 min post-infection uncover the temporal dynamics of infection-regulated genes (Tierney et al., 2012). For 21,251 mouse genes and 6,274 *Candida* genes, we found at least one RNA-seq read matched and performed statistical analyses of all time points compared to 0 min post-infection. This revealed 413 significantly deregulated genes in the mouse transcriptome and 1,068 significantly deregulated genes in the fungal transcriptome. The number of deregulated mouse genes was increasing from time point to time point: 45 genes after 30 min, 169 genes after 60 min, 239 genes after 90 min, and 300 genes after 120 min). Similar to mouse, the number of significant *Candida* genes was also increasing with 314 genes after 30 min, 316 genes after 60 min, 432 genes after 90 min, and 744 genes after 120 min post-infection (see **Figures 3A,B**). Interestingly, significantly deregulated genes in mouse were mainly upregulated genes, at a ratio 5:1. In contrast, the significant genes in *Candida* showed almost the same number of up- and downregulated genes.

With the identified deregulated genes in *C. albicans* and *M. musculus*, we generated a set of infection-associated genes each for the fungal pathogen and the mammalian host. With these sets as a positive list, deregulated genes were significantly enriched in the final refined network compared to the primary inferred mouse–*Candida* PPI network (see **Figures 3C,D**). For the predicted mouse interactors, the localization-based filtering step did not show a significant enrichment in contrast to the functional refinement. Due to the small number of interactors (12 of 41) in the refined network, the functional refinement step did not show a significant enrichment for the predicted *Candida* interactors. While the deregulated mouse genes were significantly enriched by the interolog-based inference step, the significant *Candida* genes were significantly depleted. This showed that for a vast number of pathogen-related genes in *Candida*, there were no interologous interactions found in the template networks.

#### Pathogenicity-Associated Genes are Enriched in Resulting Host–Fungi Networks

Since databases even specialized on PHI contained very few PPI between human and fungal (mainly *S. cerevisiae*) pathogens [e.g., HPIDB comprised 126 host–fungal PPIs], we extracted all human genes interacting with Archaean (0.03%), protozoan (0.3%), fungal (3.6%), or bacterial (96.1%) pathogen genes. Viral interactions were not included in our dataset as these interactions are mainly intracellular. This yielded pathogenicity-associations for 3,419 of the 20,688 protein-coding human genes which translates to a fraction of 16.5%. In contrast to the large number of human interactors, there were only 32 PHI mouse genes in the database. Because of the small number of mouse genes interacting with different pathogens, we focused on human as host.

**FIGURE 4:**
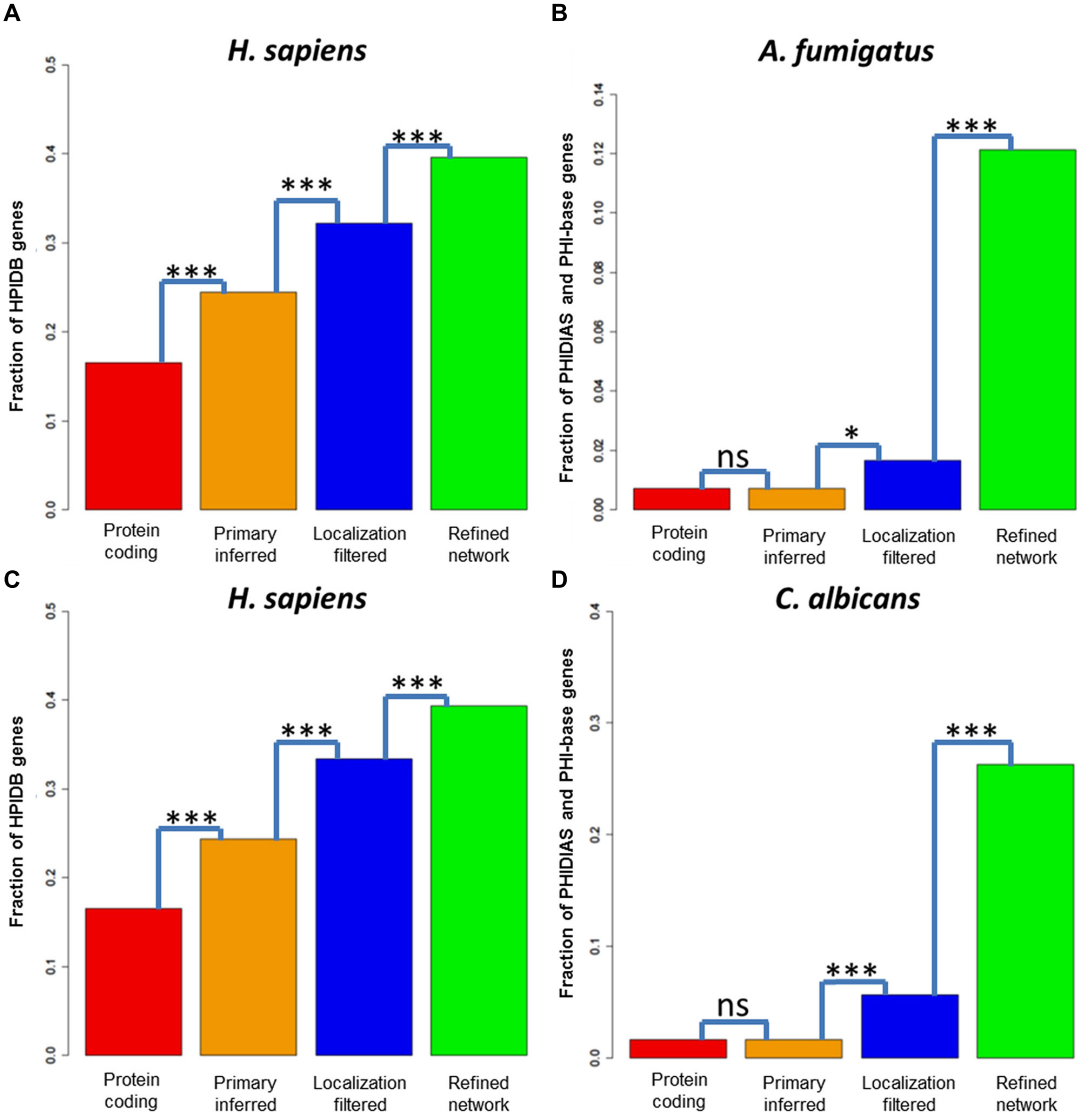
**Pathogenicity-associated genes in predicted host and fungi interactors**. Fraction of pathogenicity-associated genes in the sets of protein-coding genes, the primary inferred, the localization-filtered and the biological process refined interactors of **(A)** *H. sapiens* **(B)** *Aspergillus fumigatus* **(C)** *H. sapiens* **(D)** *C. albicans*. A test for enrichment of pathogenicity-associated genes in the interactor sets after the primary inference, localization and functional refinement step (Fisher exact test,*p < 0.05; ***p < 0.001).

The network inference step with *A. fumigatus* as fungal pathogen enriched the pathogenicity-associated genes significantly to a fraction of 24.4% (see **Figure 4A**). Further, the localization filtering for potential host–fungal interfaces also enriched the pathogenicity-relevant genes significantly to a fraction of 32.2%. At last, the refinement step for interactors associated to pathogenicity-relevant processes enriched the fraction to 39.5% (see **Figure 4A**). For human interactors with *C. albicans* as pathogen, we observed a similar enrichment of pathogenicity-associated genes from the protein-coding genes (16.5%) over the inferred (24.4%) and the localization-filtered (33.3%) to the pathogenicity-associated process refined (39.3%) interactors (see **Figure 4C**).

Due to the lack of knowledge about *C. albicans* and *A. fumigatus* PHIs, we exploited information of the databases PHI-base (Winnenburg et al., 2008) and PHIDIAS (Xiang et al., 2007) about experimentally validated virulence-associated genes. For the fungal pathogen *A. fumigatus*, we found 39 pathogenicity-associated genes in PHI-base and 29 genes in PHIDIAS (with an overlap of 14 genes), while for *C. albicans* 128 genes were found in PHI-base and 100 genes in PHIDIAS (with an overlap of 35 genes).

For the fungal pathogen *A. fumigatus*, the fraction of pathogenicity-relevant genes (0.7%) interacting with human genes was not significant for the interolog-based inference step (0.7%), weakly significant for the localization filtering step (1.7%) and strongly significant for the infection-relevant process refinement step (12.1%), (see **Figure 4B**). Similarly, the fraction of pathogenicity-associated genes (1.6%) did not increase significantly via the interolog-based inference step (1.6%), but strongly significant for the localization filtering step (5.6%) and strongly significant for the infection-relevant process refinement step (26.3%), (see **Figure 4D**).

#### Cells Involved in Immune Response and Tissues Typically Infected by Fungal Pathogens in the Resulting Host–Fungi PPI Networks

The tissue enrichment of refined *H. sapiens* interactors with either *C. albicans* or *A. fumigatus* and the primary *H. sapiens* interactors yielded several fungal infection relevant tissues (see Supplementary Tables S5 and S6). For both pathogens the cell type “Platelet” was most significantly enriched. This correlates with an investigation that attachment of platelets to fungal surfaces induced morphological changes in *Candida* spp., such as loosening of discoid shape, generation of pseudopodia, and flattened structure (Robert et al., 2000). Similar findings were described for *A. fumigatus* showing that hyphal growth is likely to induce platelet activation (Rodland et al., 2010). More in particular, certain cell wall components of *A. fumigatus*, e.g., melanin and galactosaminogalactan were involved in platelet activation while hydrophobin prevented recognition from the host immune system (Rambach et al., 2015). Besides platelets, the immune system-associated terms “B-cell lymphoma,” “T-cell,” “B-cell,” “Leukemic T-cell,” and “Peripheral blood lymphocyte” were significantly enriched. Furthermore, we observed significantly enriched tissue terms of typical environments of *Aspergillus* and *Candida* infections in the human body (“Lung,” “Epithelium,” “Blood,” “Brain,” and “Skin”). Interestingly, the tissues “Urinary bladder” and “Cervix” but also “Bone” were significantly enriched (see Supplementary Table S6).

### Exploring the Refined Host–Fungi PPI Networks

**FIGURE 5:**
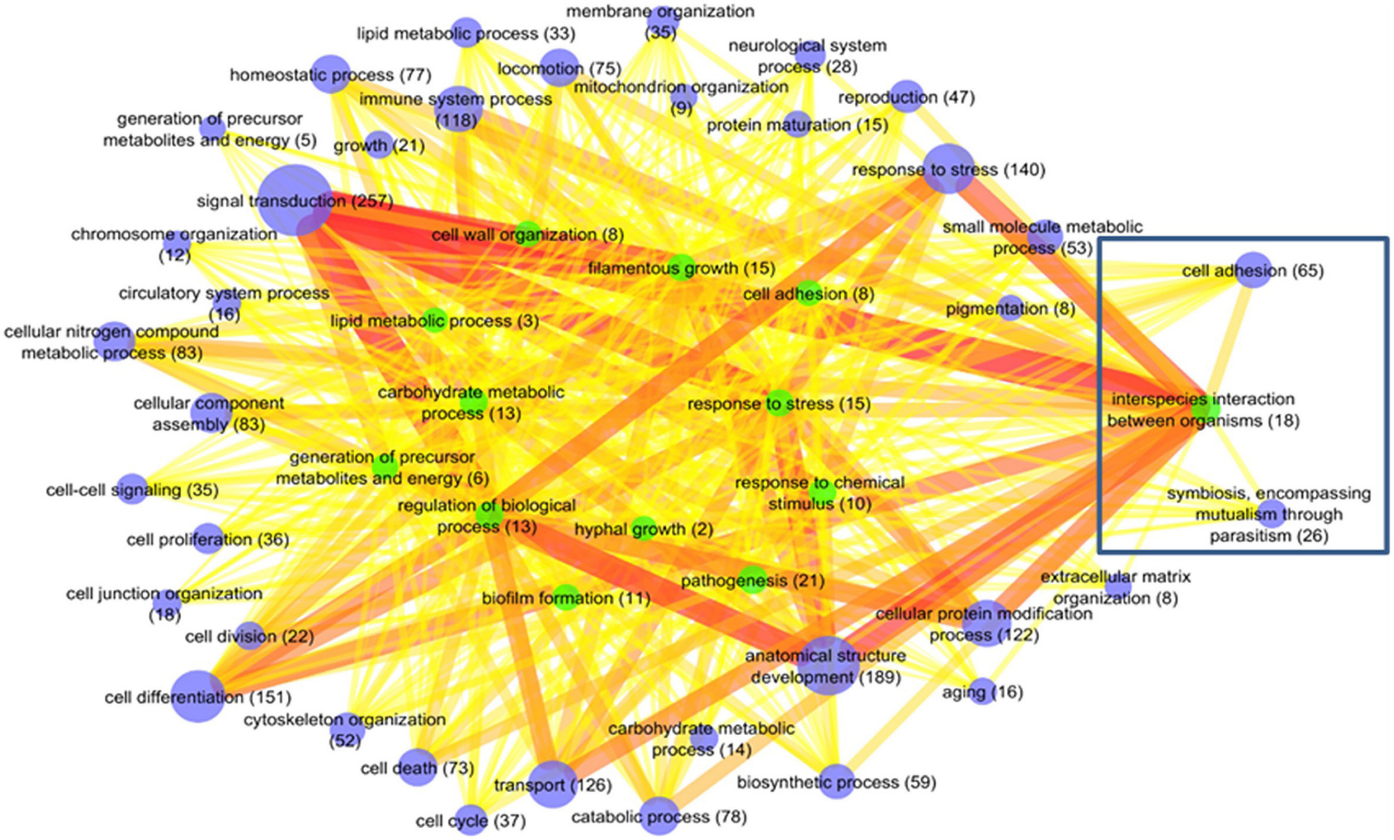
***Mus musculus*–*C. albicans* network of functional GO terms.** Nodes represent GO slim terms and edges depict interactions between host and fungal genes belonging to the particular GO slim terms. The node size denotes the number of genes in each GO slim term. The edge width and edge color correspond to the number of interactions between the connected nodes from thin/yellow to thick/red representing low to high interaction degrees. Fungal GO slim terms are visualized by green nodes and murine GO slim terms by blue nodes. The top 10% fungal pathogen interactors with the most interactions were removed from the network visualization to improve readability of the figure. The box shows the subnetworks that are evaluated in more detail.

To obtain an overview of the resulting refined networks, we visualized the interactors grouped by the functional GO slim biological process classes. Hence, the nodes represent GO slim terms and edges depict interactions between host and fungal genes belonging to the particular GO slim terms. Since the refined networks were dominated by few fungal interactors showing very high numbers of interactions, the top 10% of high degree fungal interactors (*C. albicans*: HSP90, UBI4, SSB1, SSA2, CaJ7_0234; *A. fumigatus*: glyceraldehyde-3-phosphate dehydrogenase GpdA, molecular chaperone and allergen Mod-E/Hsp90/Hsp1, 14-3-3 family protein ArtA) were removed from the network visualizations to improve clearness and readability of the figures (see **Figure 5** and Supplementary Figure S2).

In the *M. musculus* (330 interactors) and *C. albicans* (37 interactors) network, “signal transduction,” “anatomical structure development,” “cell differentiation,” “response to stress,” and “transport” represent the host GO slim terms consisting of the largest numbers of genes. For *Candida*, the terms comprising of the most interactors were “pathogenesis,” “interspecies interaction between organisms,” “filamentous growth,” “response to stress,” and “carbohydrate metabolic process.” As expected, large murine GO slim terms frequently interact with large fungal GO slim terms (e.g., 795 interactions between “signal transduction” and “regulation of biological process” or 767 between “signal transduction” and “interspecies interaction between organisms”; see **Figure 5**).

In the refined PPI network with *H. sapiens* (317 interactors) and *A. fumigatus* (30 interactors), “signal transduction,” “transport,” “cellular nitrogen compound metabolic process,” “response to stress,” and “catabolic process” represent the host GO slim terms consisting of the largest numbers of genes. For *Aspergillus*, the terms comprising of the most interactors were “pathogenesis,” “response to stress,” “carbohydrate metabolic process,” “response to chemical stimulus,” and “cell cycle.” Like for the mouse–*Candida* PPI network, large host GO slim terms frequently interact with large *Aspergillus* GO slim terms (e.g., 381 interactions between “signal transduction” and “pathogenesis” or 298 between “transport” and “pathogenesis”; see Supplementary Figure S2).

#### Mouse–*Candida* Subnetworks Contain Infection Related Interaction Candidates

**FIGURE 6:**
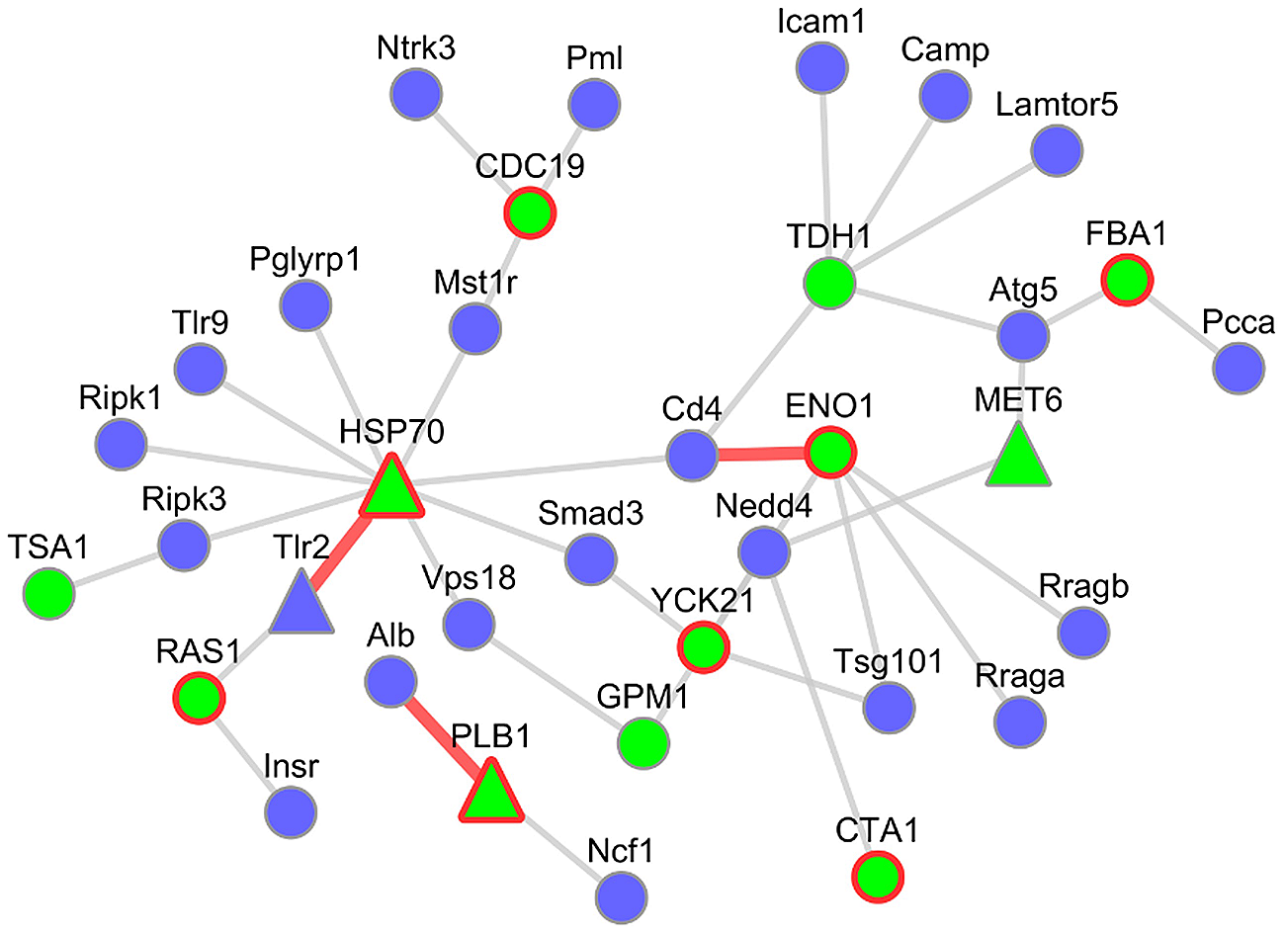
**Host–pathogen PPI subnetwork between *M. musculus* and *C. albicans.*** This subnetwork comprises host interactors annotated as “symbiosis, encompassing mutualism through parasitism” and pathogen interactors annotated as “interspecies interaction between organisms.” Blue nodes represent host interactors and green nodes fungal interactors. Nodes with a red border showed evidence for virulence contribution (PHIDIAS, PHI-base, and CGD). A triangular shape depicts infection-regulated genes of the analyzed mouse–*Candida* RNA-seq data. Interactions highlighted by red edges are described in more detail.

To investigate these networks in more detail, we focused on the subnetwork between the pathogenicity-relevant GO slim terms “symbiosis, encompassing mutualism through parasitism” and “interspecies interaction between organisms” (see **Figure 6**). This subnetwork consists of 37 interactions with 23 murine interactors out of which one was infection regulated, and 12 *C. albicans* interactors of which three were infection regulated and eight supported by PHIDIAS/PHI-base evidence. For several interaction candidates, we found additional evidence in a literature research.

##### ENO1 and Cd4

One of those is the *Candida* ENO1 (2-phospho-D-glycerate-hydrolyase) interacting with the mouse Cd4 (CD4 antigen). The Cd4 molecule is an important co-receptor of T-lymphocytes that interacts with MHC Class II antigens. It is expressed in several immune cell types and initiates or augments the early phase of T-cell activation (Gibbings and Befus, 2009). The predicted interaction partner on the pathogen side, ENO1, is not only a key component of glycolysis (Sundstrom and Aliaga, 1992), but is also an immunodominant antigen circulating in the bloodstream of patients with disseminated *Candida* infections (Sundstrom and Aliaga, 1992) and a highly immunogenic protein in *Candida*-infected mice (Pitarch et al., 2001). Moreover, ENO1 was identified as an antigen that induced protective IgG2a antibody isotype in the sera from vaccinated animals and is thus considered a potential candidate for a vaccine (Fernandez-Arenas et al., 2004). Although ENO1 is primarily a cytoplasmic protein, it has also been discovered to be an integral cell wall protein (Angiolella et al., 1996). Interestingly, another infection-associated interaction partner in the refined PHI network is plasminogen, the inactive precursor of plasmin which has been described to facilitate the invasion of the host tissues (Jong et al., 2003).

##### PLB1 and Alb

A further interesting candidate is the interaction between the murine Alb (Albumin) and *Candida* PLB1 (Phospholipase B). It has been described that the extracellular part of PLB1 is required for wild-type virulence of *Candida* in a mouse model of systemic infection (Ghannoum, 1998), possibly related to its secretion from the hyphal tip during the infection process (Ghannoum, 2000). PLB1 can penetrate wild-type host cells by lysing the plasma membrane (Park et al., 2013). Its interaction partner on the host side, Albumin, was shown to bind to germ-tubes (Page and Odds, 1988) and to inhibit the binding of PLB1 to its substrate (Reisfeld et al., 1994). In the transcriptome data set of murine innate immune cells infected by *C. albicans*, PLB1 was significantly deregulated.

##### HSP70 and Tlr2

Heat shock proteins have been described to play a role during fungal infection (Lopez-Ribot et al., 1996). Our results predict an interaction between the *Candida* HSP70 (Heat shock protein 70) and the murine Tlr2 (Toll-like receptor 2). The *Candida* HSP70 was detected on the surface of both yeast form and hyphal form cells (Urban et al., 2003) and is a member of a protein family which represents highly conserved immunodominant antigens (La Valle et al., 1995). *In vitro* studies showed that a *Candida* HSP70 mutant caused less damage to endothelial cells and oral epithelial cell lines (Sun et al., 2010). On the host side Tlr2 plays an important role in the activation of the innate immunity: It belongs to the family of pattern recognition receptors (PRRs) which are involved in the recognition of pathogen-associated molecular patterns (PAMPs), (Oliveira-Nascimento et al., 2012). Interestingly, the transcripts of both interaction partners were differentially upregulated during the infection process in the mouse–*Candida* dual RNA-seq experiment.

The mouse–*Candida* subnetwork of the host GO slim term “cell adhesion” and the fungal GO slim term “interspecies interaction between organisms” consisted of 98 interactions with 54 murine interactors (two significantly deregulated) and 16 *C. albicans* interacting partners (4 significantly deregulated, 11 supported by PHIDIAS/PHI-base evidence; see Supplementary Figure S3).

##### PLB1 and App

For the fungal PLB1 (Phospholipase B), we discovered a further potential interaction to the murine App [amyloid beta (A4) precursor protein]. APP is a cell surface receptor that mediates cell–cell and cell-matrix adhesion (Stahl et al., 2014) and is cleaved by secretases to form a number of peptides. Although, the human APP is primarily known for its role in Alzheimer’s Disease (Gorevic et al., 1986), some of the App peptides have antibiotic activity against at least eight common and clinically relevant microorganisms, i.e., Gram-negative, Gram-positive bacteria, and the yeast *C. albicans* with the latter being the most sensitive (Soscia et al., 2010).

##### CDC19 and Egfr

We also found evidence for a very interesting interaction between the fungal CDC19 protein (Pyruvate kinase CDC19) and the murine Egfr protein (epidermal growth factor receptor). The fungal interactor CDC19, usually, an enzyme of the glycolysis, was found to be present on the yeast-form cell surface of *C. albicans* (Pitarch et al., 2002) and differentially expressed after 3-h co-culture with murine macrophages (Fernandez-Arenas et al., 2007). Furthermore, it is an immunogenic protein that is specifically recognized by antibodies in sera of vaccinated and of systemically *Candida*-infected mice (Pitarch et al., 2001; Thomas et al., 2006; Martinez-Lopez et al., 2008). A homozygous null mutant showed decreased virulence and filamentous growth (Binkley et al., 2014). Egfr is a transmembrane glycoprotein and receptor of the epidermal growth factor family. Egfr was shown to induce endocytosis of *C. albicans* by epithelial cells (Zhu et al., 2012). Furthermore, there is evidence for the secreted agrA (Accessory gene regulator protein A) of *Staphylococcus aureus* to bind to Egfr and activate a signal pathway in a pathogenicity-associated process (Gomez et al., 2007).

#### Examples for Interesting Human–*Aspergillus* PPIs in the Resulting Host–Fungi Network

**FIGURE 7:**
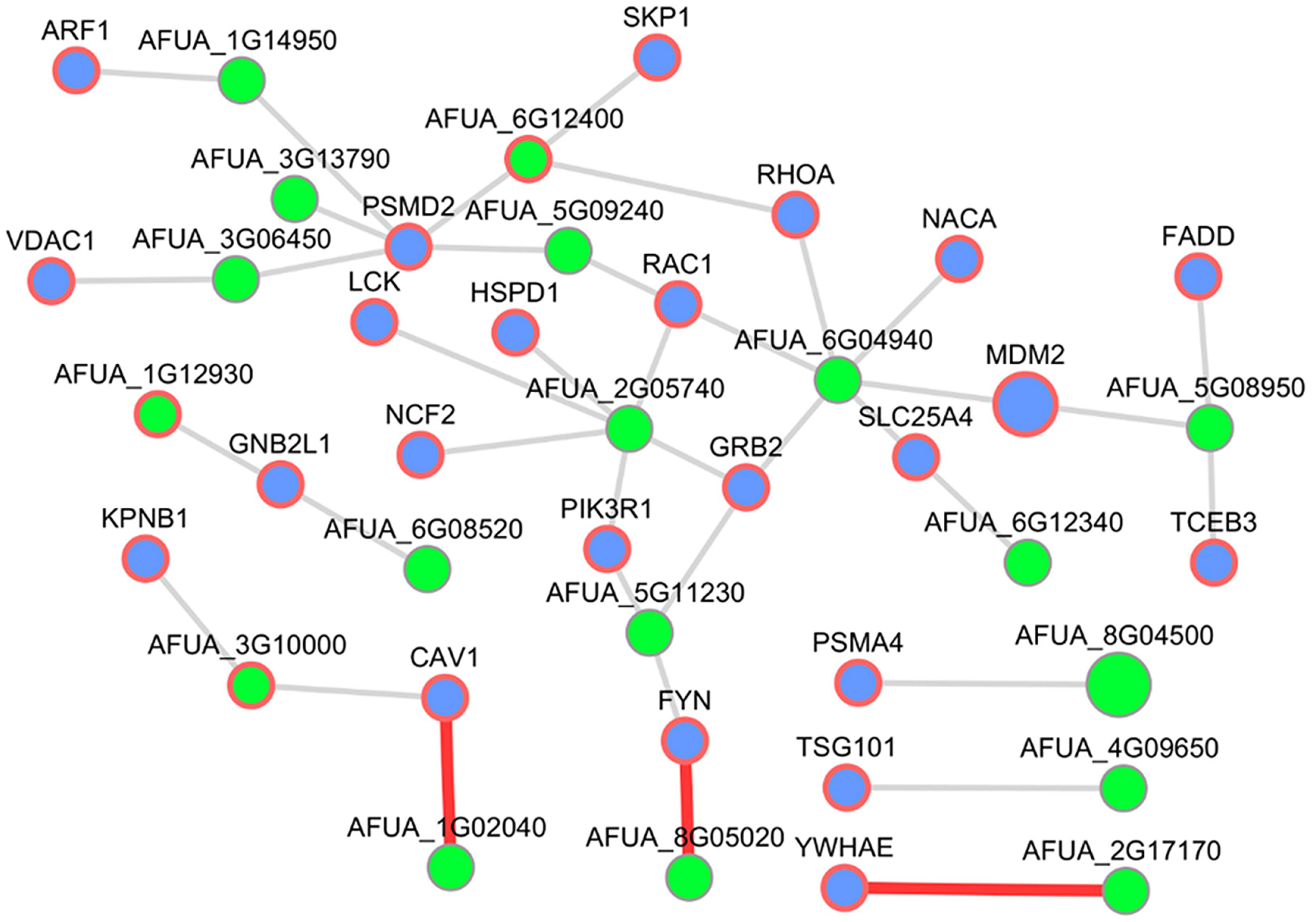
**Host–pathogen PPI subnetwork between *H. sapiens* and *A. fumigatus*.** This subnetwork comprises pathogenicity-associated (HPIDB) host interactors annotated as “symbiosis, encompassing mutualism through parasitism” and pathogen interactors annotated as “pathogenesis.” Blue nodes represent host interactors and green nodes fungal interactors. Nodes with a red border showed evidence for virulence contribution (PHIDIAS, PHI-base, and AspGD) or other host–pathogen interactions (HPIDB). Interactions highlighted by red edges were described in more detail.

Since very little is known about human–*Aspergillu*s interactions in available databases up to date, we selected the infection-relevant subnetwork of interactions between the host GO slim term “symbiosis, encompassing mutualism through parasitism” and the fungal GO slim term “pathogenesis.” To get a transparent size, we visualized only host nodes pathogenicity-associated based on HPIDB and removed the human interactor UBC (ubiquitin C) due to the high number of interactions. This subnetwork consists of 38 interactions with 23 human interactors and 18 *A. fumigatus* interacting partners (three supported by PHIDIAS/PHI-base evidence; see **Figure 7**).

##### RBE1 and CAV

The interesting interaction between the human CAV1 (caveolin 1) and the *Aspergillus* AFUA_1G02040 (Uncharacterized protein) in that subnetwork was inferred from the human template CAV1 – GLIPR2 (GLI pathogenesis-related 2) detected by affinity chromatography technology (Eberle et al., 2002). The *C. albicans* ortholog of AFUA_1G02040, RBE1 (Repressed by EFG1 protein 1), is a Pry family cell wall protein (Sohn et al., 2003) and belongs to a group of plant pathogenesis-related proteins (PR-1; Rohm et al., 2013). A homozygote null mutant of RBE1 in *Candida* showed a decreased virulence and increased sensitivity to attack by polymorphonuclear leucocytes (Rohm et al., 2013). The human CAV1 is the major structural protein in the caveolae of endothelial cells (Smart et al., 1999). It is also involved in the costimulatory signal essential for T-cell receptor (TCR)-mediated T-cell activation (Ohnuma et al., 2007) and can act as a functional receptor for CD26 in antigen representing cells (Ohnuma et al., 2004) which implies a cell surface localization.

##### CNH1 and YWHAE

In addition, we discovered another promising interaction, namely between the human YWHAE (tyrosine 3-monooxygenase/tryptophan 5-monooxygenase activation protein) – AFUA_2G17170 (Uncharacterized protein) which is an ortholog of the fungal-specific *C. albicans* Na^+^/H^+^ antiporter CNH1 (Inglis et al., 2012). Homozygous null mutants of *Candida* ortholog showed decreased virulence (Soong et al., 2000). The human YWHAE, member of the 14-3-3 protein family was co-immunoprecipitated with MHC II in B-cell exosomes (Buschow et al., 2010) and thus implying an immune response relevant function.

##### HEX1 and FYN

In the human–*Aspergillus* subnetwork, we predicted an interaction between the human FYN (FYN Proto-oncogene) and the *Aspergillus* AFUA_8G05020 (Uncharacterized protein). FYN is a membrane-associated tyrosine kinase (Morford et al., 2002) and localized in the endosome (Puertollano, 2005). Further, it plays an important role in T-cell activation (Lancki et al., 1995). The *Aspergillus* AFUA_8G05020 is a putative secreted *N*-acetylhexosaminidase (Bruns et al., 2010; Sharma et al., 2011) which is highly expressed in biofilm (Bruns et al., 2010). Furthermore, the *C. albicans* ortholog HEX1 is required for full virulence and these proteins may have a role in carbon or nitrogen scavenging (Niimi et al., 1997).

## Discussion

Even though fungal infections are clinically highly relevant and impose a substantial disease burden worldwide (Brown et al., 2012), not much data about interactions between fungal pathogens and the human host on a molecular level are currently available. In our study, a comprehensive search of publically available PHIs (Kumar and Nanduri, 2010) yielded only a small number of reported host–fungi PPIs. Also, thorough searches of all major PPI databases for cross-species interaction revealed only a few fungal candidates. This obvious sparseness of established experimental data on molecular host–fungal interactions generates an important and valuable research challenge for novel PHI prediction approaches. While *in silico* methods for the prediction of molecular interactions between host and pathogenic organisms have been receiving growing attention in the last years, the main focus still lays on viral and bacterial pathogens (Zhou et al., 2013a), and fungal species have only been sparsely investigated. To our knowledge, a thorough systematic prediction and analysis of *A. fumigatus* and *C. albicans* interactions with the human and murine host has not been performed so far.

In this study, we developed and examined an interolog-based method for the prediction of fungal–host interactions. We focused our investigation on two of the most clinically relevant fungi *C. albicans* and *A. fumigatus*. Since murine mouse models have become an invaluable tool in medical research, we also investigated interactions between these fungi and *M. musculus* in addition to the human host. As the primary objective of our study was to attain a comprehensive catalog of high quality PHI predictions, we used an extended dual species template approach which is based on human and yeast, the two best studied species for PPI network. By this we effectively made use of the majority of all publically available PPI data. Compared to simple approaches relying on the yeast template only, we created a considerably enhanced prediction space, in particular on the host side, which increases the set of interactors for human and mouse by over 200%.

A potential limitation of interspecies interolog approaches is the fact that the prediction space is confined to interactions between proteins with orthologs counterparts in the source network on either side. Hence, basing a prediction approach exclusively on the yeast network could lead to a bias toward ancient well conserved proteins and exclude less conserved ‘newer’ genes and pathways. These could include also host-specific genes such as those involved in novel adaptive immune responses. The inclusion of the human template network partially alleviates those effects as, at least on the host side, no basal orthology relationship is required. Our results suggest that a large and in particular human based template network is a key prerequisite for the prediction of functionally more relevant interactions.

Nevertheless, homology based approaches are known to be prone to produce overpredictions, since, in the first step, pairwise interactions are inferred between all homologs regardless of their cellular function or localization. Indeed, the predicted interaction partners on either side may in fact have little opportunity to physically interact with each other. This applies in particular to proteins which are expressed exclusively in the intracellular compartment and might thus have little opportunity to interact with the predicted host/pathogen counterpart. Although we applied a rigorous filtering cascade to exclude many (99.4%) of these potentially spurious interaction predictions, we noted that many proteins are expressed in various subcellular compartments. In particular, numerous intracellular proteins can shuttle to the membrane compartment or even be secreted. To narrow down this set of ‘potentially physically possible’ predictions, we focused on interactors involved in pathways which play important roles during cellular infection processes.

Enrichment analyses using independent data (Xiang et al., 2007; Winnenburg et al., 2008; Kumar and Nanduri, 2010) revealed a clearly increasing fraction of virulence and pathogenicity-associated genes during the refinement process, suggesting a large set of functionally relevant interactions among the predictions. Moreover, on the host side we found an enrichment of genes which are expressed in tissues that are specifically affected by fungal infections, e.g., activation of platelets by *A. fumigatus* (Rodland et al., 2010) and *C. albicans* (Robert et al., 2000).

Our extended interolog-based approach assembled a large catalog of PHIs. As this homology based approach is tied to the template interaction network, it is confined to the set of reported physical PPIs and thus also inherits the set false positives from the template network. Therefore, an interesting complementary approach would be the investigation of an approach based on domain–domain interactions (Zhou et al., 2013b). This would eliminate the necessity of homology for the predicted interactors, as it only requires the presence of the interacting domains. Thus, it can be expected to yield a complementary dataset. Similarly, inference methods based on the correlated gene expression in host and pathogen (e.g., measured over an infection time course), are an interesting approach which could be further explored, in combination with and in comparison to the interolog approach (Wang et al., 2013; Weber et al., 2013; Schulze et al., 2015). Certainly, the assembly of large PHI networks establishes an ample hypotheses space as a basis which can be exploited by advanced methods of integrative network analysis (Dittrich et al., 2008; Beisser et al., 2012), for which a large number of approaches have been established in the last years. Here, further development is needed to extend these approaches to the simultaneous analysis of the complex connected host and pathogen networks. Albeit, technically not trivial, it is unquestionably a worthwhile task as it holds the potential to link subcellular response pathways between host and pathogen during the dynamics of the infection process.

## Conflict of Interest Statement

The authors declare that the research was conducted in the absence of any commercial or financial relationships that could be construed as a potential conflict of interest.

## Footnotes

1 wiki.thebiogrid.org/doku.php/statistics

2 http://www.mechanobio.info/

3 http://www.molecularconnections.com

4 http://www.ucl.ac.uk/functional-gene-annotation/cardiovascular

5 http://software.sbc.su.se/cgi-bin/request.cgi?project=inparanoid
