# Supplementary material for: Integrated inference and evaluation of host–fungi interaction networks
Source: Front Microbiol. 2015 Aug 4;6:764. doi: 10.3389/fmicb.2015.00764 (PMC4523839; doi:10.3389/fmicb.2015.00764)
Supplement: Supplementary file 1 [file Data_Sheet_1.PDF]

Frontiers in Microbiology

Original Research
03/04/2015

Supplementary Material:

Integrated inference and evaluation of host-fungi interaction networks

Christian W. Remmele[1], Christian Luther[1], Johannes Balkenhol[1], Thomas Dandekar[1], Tobias Müller[1], Marcus T. Dittrich[1,2]*,

[1] Department of Bioinformatics, University of Würzburg, Würzburg, Germany
[2] Department of Human Genetics, University of Würzburg, Würzburg, Germany

Figure S1:

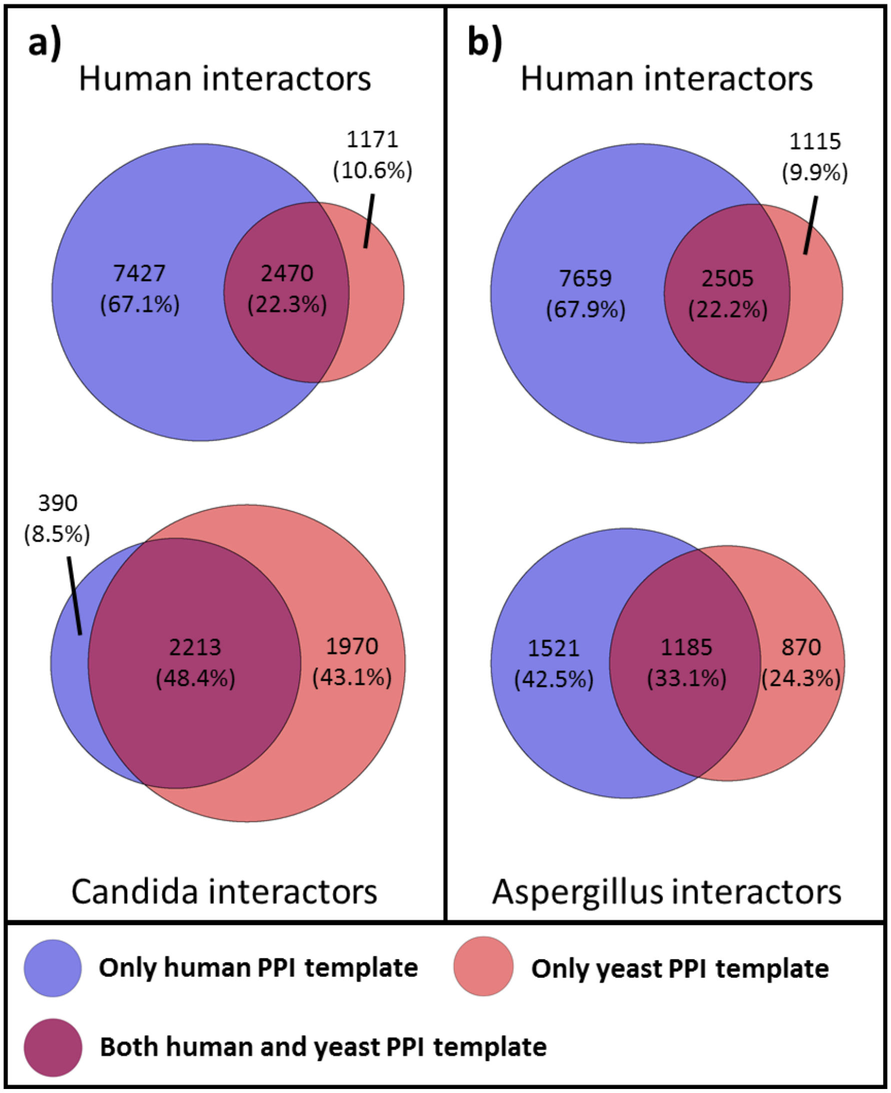

Figure S1. Influence of the template networks on the predicted a) human - Candida network b) human - Aspergillus network. The color of the circle denotes the template network, the interactors originated from.

Figure S2:

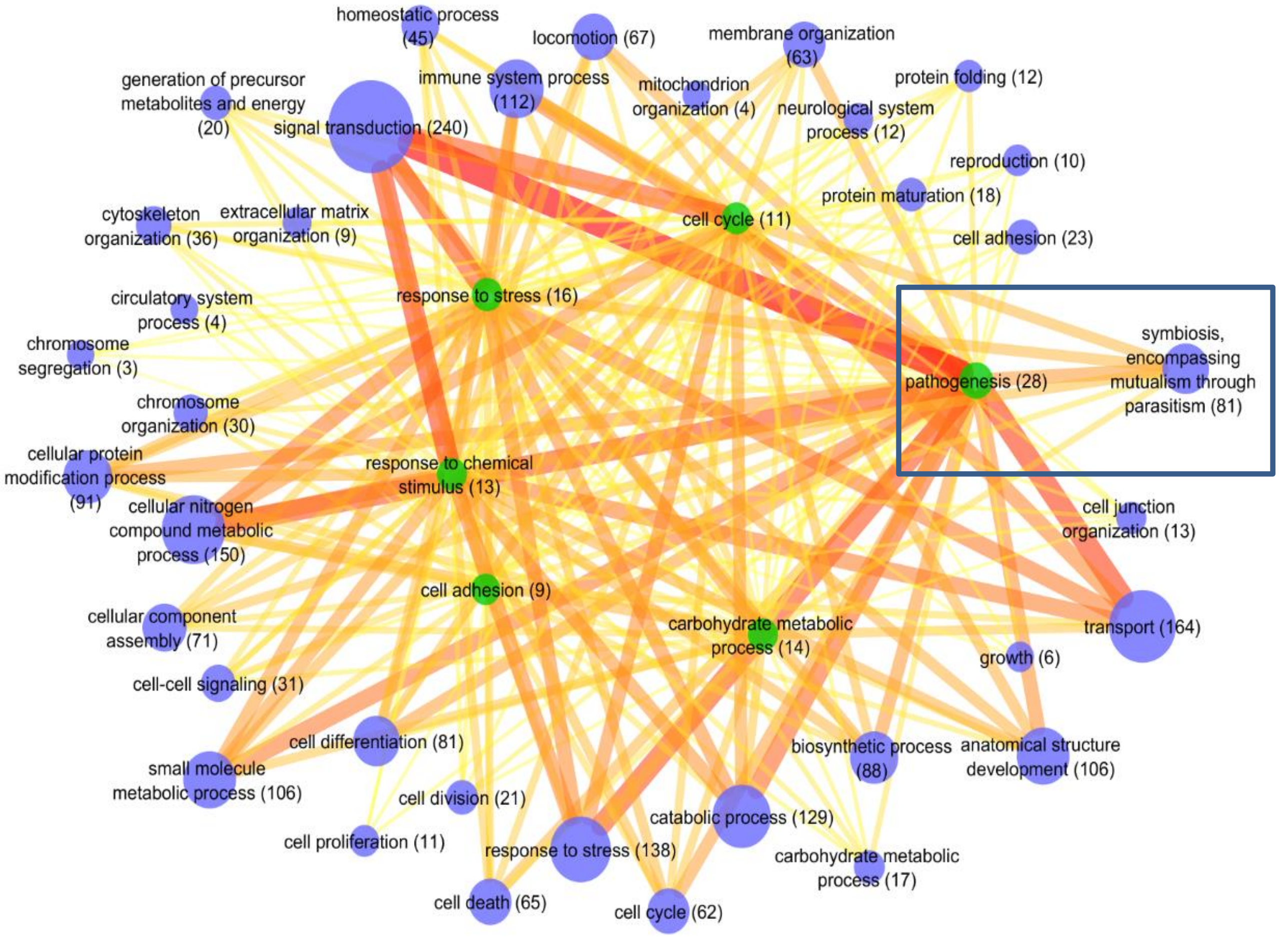

Figure S2. Refined network of functional GO terms with *H. sapiens* and *A. fumigatus*. Nodes represent GO slim terms and edges depict interactions between host and fungal genes belonging to the particular GO slim terms. The node size denotes the number of genes in each GO slim term. The edge width and edge color correspond to the number of interactions between the connected nodes from yellow to red representing low to high interaction degrees. Fungal GO slim terms are visualized by green nodes and human GO slim terms by blue color. The top 10% fungal pathogen interactors with the most interactions were removed from the network visualizations to improve readability of the figure. The box shows the subnetwork that is evaluated in more detail.

Figure S3:

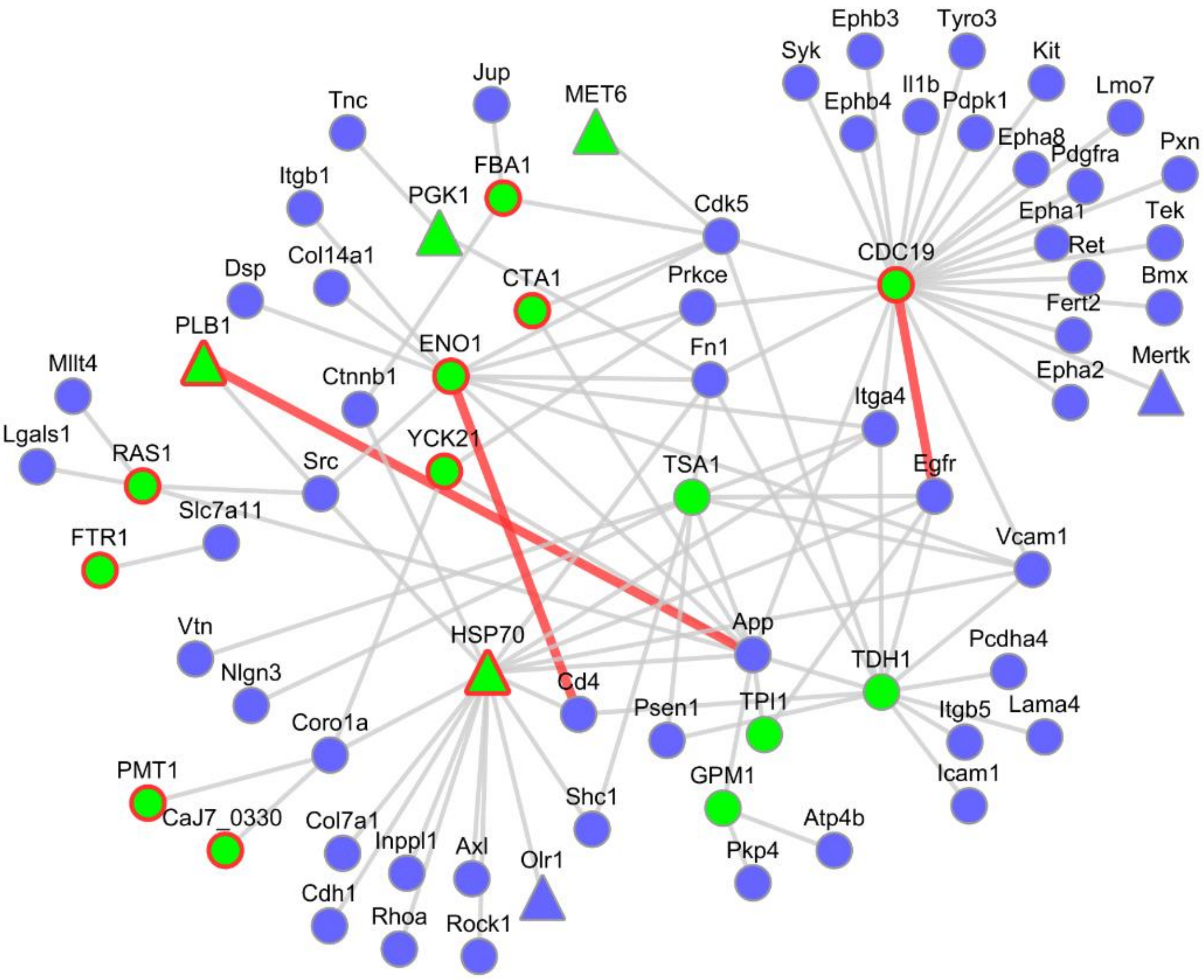

Figure S3. Host-pathogen PPI subnetwork between *M. musculus* and *C. albicans.* This subnetwork comprises host interactors annotated as "cell adhesion" and pathogen interactors annotated as "interspecies interaction between organisms". Blue nodes represent host interactors and green nodes fungal interactors. Nodes with a red border showed evidence to be involved in virulence or host-pathogen interactions (PHIDIAS, PHI-base, and CGD). A triangular shape depicts infection regulated genes in the Candida-mouse infection experiment. Interactions highlighted by red edges are described in more detail.

Table S1. Orthology relationship of investigated species. First column shows the investigated species and the second column shows the template networks species. Orthology relationships are described in different perspectives. Column three shows the seed othologs' relation establishing ortholgous groups, whereas column four counts include additional paralogs to orthologous groups. Latter two columns show orthologs including paralogs for each species.

Target species	Template species	Orthologous groups	Orthologous pairs	Orthologs in target species (Entrez GI)	Orthologs in template species (Entrez GI)	Source of orthology information
H. sapiens	*S. cerevisiae*	2,040	4,538	3,645	2,388	Inparanoid8
M. muscuslus	*H. sapiens*	15,849	18,030	16,582	16,417	Inparanoid8
M. muscuslus	*S. cerevisiae*	2,003	4,322	3,556	2,341	Inparanoid8
C. albicans	*H. sapiens*	2,059	5,334	2,687	3,770	Inparanoid8
C. albicans	*S. cerevisiae*	3,435	4,941	4,220	3,864	CGD
A. fumigatus	*H. sapiens*	2,451	5,580	2,808	4,277	Stand-alone
A. fumigatus	*S. cerevisiae*	1,923	2,551	2,083	2,264	AspGD

Table S2. Primary inferred host-fungi PPI networks. Overview on the number of host and pathogen interactors as well as the number of host-pathogen interactions for human-Candida, human-Aspergillus, mouse-Candida and mouse-Aspergillus.

Host species	Pathogen species	Number of host interactors	Number of pathogen interactors	Number of host-pathogen interactions
H. sapiens	*C. albicans*	11,068	4,573	353,866
H. sapiens	*A. fumigatus*	11,279	3,576	213,518
M. musculus	*C. albicans*	10,358	4,575	337,686
M. musculus	*A. fumigatus*	10,560	3,576	205,089

Table S3. Significantly enriched GO terms in human genes of the *H. sapiens-A. fumigatus* network in interactions inferred a) only by the human template network b) only by the yeast template network c) by both human and yeast template network. Only GO groups with significant enrichment ($p < 0.1$) after multiple testing correction (Hochberg and Benjamini, 1990) were reported. For visualization of the gene sets see figure 2.

a) Inferred only by the human template network					
Category	GO Term	Number of genes	%	Fold Enrichment	Adjusted p-value
GOTERM_MF_2	GO:0005515~protein binding	4505	59.65	1.09	1.89E-61
GOTERM_MF_2	GO:0004871~signal transducer activity	906	12.00	1.32	6.31E-60
GOTERM_BP_2	GO:0048856~anatomical structure development	1402	18.56	1.25	2.48E-57
GOTERM_BP_2	GO:0007275~multicellular organismal development	1531	20.27	1.23	1.25E-55
GOTERM_BP_2	GO:0048869~cellular developmental process	969	12.83	1.26	8.15E-41
GOTERM_CC_2	GO:0044421~extracellular region part	418	5.53	1.39	1.62E-38
GOTERM_BP_2	GO:0009653~anatomical structure morphogenesis	710	9.40	1.29	1.70E-37
GOTERM_BP_2	GO:0007155~cell adhesion	342	4.53	1.43	1.74E-37
GOTERM_BP_2	GO:0050789~regulation of biological process	3442	45.58	1.09	3.61E-37
GOTERM_BP_2	GO:0050794~regulation of cellular process	3291	43.58	1.09	5.40E-33
GOTERM_BP_2	GO:0051239~regulation of multicellular organismal process	573	7.59	1.30	1.50E-31
GOTERM_BP_2	GO:0009605~response to external stimulus	510	6.75	1.31	7.17E-29
GOTERM_BP_2	GO:0048519~negative regulation of biological process	1127	14.92	1.19	6.52E-29
GOTERM_CC_2	GO:0005615~extracellular space	325	4.30	1.37	8.60E-28
GOTERM_BP_2	GO:0050793~regulation of developmental process	433	5.73	1.32	1.33E-26
GOTERM_BP_2	GO:0048518~positive regulation of biological process	1205	15.96	1.17	5.74E-26
GOTERM_BP_2	GO:0048523~negative regulation of cellular process	1031	13.65	1.18	3.54E-24
GOTERM_BP_2	GO:0048522~positive regulation of cellular process	1101	14.58	1.17	2.20E-21
GOTERM_BP_2	GO:0006955~immune response	340	4.50	1.32	2.21E-20
GOTERM_BP_2	GO:0007154~cell communication	414	5.48	1.28	3.29E-20
GOTERM_BP_2	GO:0032879~regulation of localization	386	5.11	1.30	3.37E-20
GOTERM_BP_2	GO:0009790~embryonic development	355	4.70	1.29	1.08E-17
GOTERM_BP_2	GO:0048583~regulation of response to stimulus	290	3.84	1.32	3.96E-17
GOTERM_BP_2	GO:0007389~pattern specification process	163	2.16	1.40	6.77E-15
GOTERM_BP_2	GO:0007610~behavior	256	3.39	1.31	7.66E-15
GOTERM_BP_2	GO:0003008~system process	540	7.15	1.21	1.60E-14
GOTERM_BP_2	GO:0006928~cell motion	290	3.84	1.29	4.70E-14
GOTERM_BP_2	GO:0006950~response to stress	910	12.05	1.15	1.11E-13
GOTERM_BP_2	GO:0008219~cell death	449	5.95	1.22	1.62E-13
GOTERM_BP_2	GO:0048870~cell motility	195	2.58	1.34	3.31E-13
GOTERM_BP_2	GO:0051674~localization of cell	195	2.58	1.34	3.31E-13
GOTERM_CC_2	GO:0009986~cell surface	194	2.57	1.33	2.33E-12
GOTERM_BP_2	GO:0051094~positive regulation of developmental process	186	2.46	1.34	1.65E-12
GOTERM_MF_2	GO:0004857~enzyme inhibitor activity	151	2.00	1.37	7.11E-12
GOTERM_CC_2	GO:0031012~extracellular matrix	126	1.67	1.40	4.16E-12

GOTERM_BP_2	GO:0065008~regulation of biological quality	783	10.37	1.15	2.05E-12
GOTERM_BP_2	GO:0040012~regulation of locomotion	138	1.83	1.39	5.17E-12
GOTERM_BP_2	GO:0002682~regulation of immune system process	231	3.06	1.29	1.14E-11
GOTERM_CC_2	GO:0042995~cell projection	397	5.26	1.21	2.06E-11
GOTERM_BP_2	GO:0051093~negative regulation of developmental process	166	2.20	1.34	2.48E-11
GOTERM_MF_2	GO:0003700~transcription factor activity	562	7.44	1.17	1.07E-10
GOTERM_BP_2	GO:0048584~positive regulation of response to stimulus	140	1.85	1.36	2.96E-10
GOTERM_BP_2	GO:0051240~positive regulation of multicellular organismal process	154	2.04	1.33	7.86E-10
GOTERM_CC_2	GO:0045211~postsynaptic membrane	72	0.95	1.47	1.54E-09
GOTERM_BP_2	GO:0022414~reproductive process	389	5.15	1.20	9.13E-10
GOTERM_BP_2	GO:0032989~cellular component morphogenesis	241	3.19	1.25	2.63E-09
GOTERM_BP_2	GO:0048646~anatomical structure formation involved in morphogenesis	211	2.79	1.27	3.23E-09
GOTERM_BP_2	GO:0002684~positive regulation of immune system process	138	1.83	1.34	3.78E-09
GOTERM_BP_2	GO:0030030~cell projection organization	213	2.82	1.26	7.74E-09
GOTERM_CC_2	GO:0044420~extracellular matrix part	65	0.86	1.47	1.82E-08
GOTERM_BP_2	GO:0065009~regulation of molecular function	571	7.56	1.15	1.98E-08
GOTERM_CC_2	GO:0016020~membrane	2597	34.39	1.05	3.91E-08
GOTERM_BP_2	GO:0001775~cell activation	177	2.34	1.28	3.58E-08
GOTERM_MF_2	GO:0016563~transcription activator activity	297	3.93	1.20	1.44E-07
GOTERM_BP_2	GO:0042330~taxis	84	1.11	1.40	8.01E-08
GOTERM_MF_2	GO:0003712~transcription cofactor activity	268	3.55	1.21	4.50E-07
GOTERM_MF_2	GO:0060589~nucleoside-triphosphatase regulator activity	246	3.26	1.21	1.08E-06
GOTERM_BP_2	GO:0009893~positive regulation of metabolic process	559	7.40	1.13	7.54E-07
GOTERM_CC_2	GO:0044456~synapse part	131	1.73	1.29	1.20E-06
GOTERM_BP_2	GO:0051707~response to other organism	153	2.03	1.26	1.31E-06
GOTERM_BP_2	GO:0040008~regulation of growth	204	2.70	1.22	2.03E-06
GOTERM_BP_2	GO:0051128~regulation of cellular component organization	286	3.79	1.18	2.41E-06
GOTERM_BP_2	GO:0042221~response to chemical stimulus	693	9.18	1.11	2.61E-06
GOTERM_BP_2	GO:0051241~negative regulation of multicellular organismal process	94	1.24	1.34	2.79E-06
GOTERM_BP_2	GO:0051050~positive regulation of transport	128	1.69	1.28	3.33E-06
GOTERM_MF_2	GO:0016564~transcription repressor activity	226	2.99	1.20	8.95E-06
GOTERM_BP_2	GO:0034330~cell junction organization	46	0.61	1.49	4.23E-06
GOTERM_BP_2	GO:0045321~leukocyte activation	149	1.97	1.25	5.60E-06
GOTERM_BP_2	GO:0008283~cell proliferation	261	3.46	1.18	7.75E-06
GOTERM_CC_2	GO:0044425~membrane part	2229	29.52	1.05	1.34E-05
GOTERM_BP_2	GO:0051129~negative regulation of cellular component organization	102	1.35	1.30	9.88E-06
GOTERM_BP_2	GO:0040017~positive regulation of locomotion	72	0.95	1.37	9.92E-06
GOTERM_BP_2	GO:0050878~regulation of body fluid levels	79	1.05	1.35	9.81E-06
GOTERM_BP_2	GO:0009892~negative regulation of metabolic process	498	6.59	1.12	1.89E-05
GOTERM_MF_2	GO:0030246~carbohydrate binding	140	1.85	1.24	5.29E-05
GOTERM_BP_2	GO:0043062~extracellular structure organization	83	1.10	1.33	2.07E-05
GOTERM_MF_2	GO:0001871~pattern binding	71	0.94	1.35	7.05E-05

GOTERM_MF_2	GO:0008047~enzyme activator activity	193	2.56	1.19	8.58E-05
GOTERM_BP_2	GO:0002253~activation of immune response	52	0.69	1.41	4.65E-05
GOTERM_BP_2	GO:0051051~negative regulation of transport	89	1.18	1.30	5.24E-05
GOTERM_CC_2	GO:0014069~postsynaptic density	49	0.65	1.41	1.12E-04
GOTERM_BP_2	GO:0003006~reproductive developmental process	146	1.93	1.22	9.73E-05
GOTERM_BP_2	GO:0002520~immune system development	182	2.41	1.19	1.07E-04
GOTERM_BP_2	GO:0050817~coagulation	59	0.78	1.36	1.53E-04
GOTERM_BP_2	GO:0007565~female pregnancy	61	0.81	1.34	2.26E-04
GOTERM_MF_2	GO:0042165~neurotransmitter binding	35	0.46	1.46	7.37E-04
GOTERM_BP_2	GO:0032504~multicellular organism reproduction	239	3.16	1.15	3.34E-04
GOTERM_BP_2	GO:0048609~reproductive process in a multicellular organism	239	3.16	1.15	3.34E-04
GOTERM_BP_2	GO:0051130~positive regulation of cellular component organization	115	1.52	1.23	3.59E-04
GOTERM_MF_2	GO:0060090~molecular adaptor activity	47	0.62	1.38	8.09E-04
GOTERM_BP_2	GO:0009719~response to endogenous stimulus	233	3.09	1.15	3.88E-04
GOTERM_BP_2	GO:0032844~regulation of homeostatic process	71	0.94	1.30	4.50E-04
GOTERM_BP_2	GO:0019725~cellular homeostasis	227	3.01	1.15	4.52E-04
GOTERM_BP_2	GO:0002683~negative regulation of immune system process	51	0.68	1.36	5.34E-04
GOTERM_MF_2	GO:0005201~extracellular matrix structural constituent	33	0.44	1.45	0.001274233
GOTERM_BP_2	GO:0009628~response to abiotic stimulus	205	2.71	1.16	6.66E-04
GOTERM_CC_2	GO:0044463~cell projection part	120	1.59	1.21	0.001236644
GOTERM_BP_2	GO:0048585~negative regulation of response to stimulus	65	0.86	1.30	9.08E-04
GOTERM_BP_2	GO:0001503~ossification	68	0.90	1.29	9.00E-04
GOTERM_BP_2	GO:0019953~sexual reproduction	215	2.85	1.14	0.00171436
GOTERM_BP_2	GO:0051606~detection of stimulus	59	0.78	1.30	0.0018692
GOTERM_BP_2	GO:0045926~negative regulation of growth	69	0.91	1.26	0.002784797
GOTERM_BP_2	GO:0030534~adult behavior	54	0.72	1.30	0.003027333
GOTERM_BP_2	GO:0040013~negative regulation of locomotion	41	0.54	1.35	0.003367797
GOTERM_BP_2	GO:0003001~generation of a signal involved in cell-cell signaling	49	0.65	1.31	0.004919962
GOTERM_BP_2	GO:0021700~developmental maturation	63	0.83	1.26	0.005490598
GOTERM_BP_2	GO:0009607~response to biotic stimulus	196	2.60	1.13	0.005862014
GOTERM_CC_2	GO:0031252~cell leading edge	94	1.24	1.20	0.011112909
GOTERM_BP_2	GO:0009566~fertilization	35	0.46	1.36	0.007050721
GOTERM_BP_2	GO:0008037~cell recognition	28	0.37	1.42	0.00726694
GOTERM_BP_2	GO:0048771~tissue remodeling	38	0.50	1.34	0.007353078
GOTERM_BP_2	GO:0045927~positive regulation of growth	49	0.65	1.28	0.009586227
GOTERM_BP_2	GO:0007566~embryo implantation	23	0.30	1.46	0.010833203
GOTERM_MF_2	GO:0008289~lipid binding	232	3.07	1.11	0.026812579
GOTERM_MF_2	GO:0019207~kinase regulator activity	61	0.81	1.23	0.030859895
GOTERM_BP_2	GO:0019222~regulation of metabolic process	1930	25.56	1.03	0.013935862
GOTERM_BP_2	GO:0048871~multicellular organismal homeostasis	50	0.66	1.27	0.014309579
GOTERM_BP_2	GO:0007276~gamete generation	188	2.49	1.12	0.014745261
GOTERM_BP_2	GO:0050900~leukocyte migration	35	0.46	1.33	0.01566076
GOTERM_BP_2	GO:0048610~reproductive cellular process	86	1.14	1.19	0.015776754

GOTERM_BP_2	GO:0045767~regulation of anti-apoptosis	28	0.37	1.37	0.018748101
GOTERM_BP_2	GO:0032846~positive regulation of homeostatic process	21	0.28	1.45	0.019635173
GOTERM_BP_2	GO:0007632~visual behavior	24	0.32	1.40	0.022679514
GOTERM_BP_2	GO:0051705~behavioral interaction between organisms	20	0.26	1.45	0.026758493
GOTERM_BP_2	GO:0031343~positive regulation of cell killing	16	0.21	1.52	0.029183996
GOTERM_BP_2	GO:0007017~microtubule-based process	147	1.95	1.12	0.031636223
GOTERM_MF_2	GO:0050840~extracellular matrix binding	16	0.21	1.50	0.084470025
GOTERM_BP_2	GO:0002252~immune effector process	70	0.93	1.18	0.041890748
GOTERM_BP_2	GO:0031341~regulation of cell killing	18	0.24	1.44	0.04904909
GOTERM_BP_2	GO:0048589~developmental growth	51	0.68	1.21	0.05434872
GOTERM_BP_2	GO:0007416~synaptogenesis	16	0.21	1.43	0.089648777
GOTERM_BP_2	GO:0042698~ovulation cycle	38	0.50	1.23	0.091888277
**b) Inferred only by the yeast template network**					
Category	GO Term	Number of genes	%	Fold Enrichment	Adjusted p-value
GOTERM_MF_2	GO:0043167~ion binding	466	42.71	1.69	2.14E-41
GOTERM_BP_2	GO:0009058~biosynthetic process	465	42.62	1.67	2.37E-40
GOTERM_MF_2	GO:0022857~transmembrane transporter activity	126	11.55	2.57	4.72E-23
GOTERM_BP_2	GO:0055085~transmembrane transport	94	8.62	2.73	2.37E-18
GOTERM_MF_2	GO:0003676~nucleic acid binding	365	33.46	1.47	1.07E-17
GOTERM_BP_2	GO:0006807~nitrogen compound metabolic process	426	39.05	1.40	3.39E-17
GOTERM_BP_2	GO:0044238~primary metabolic process	637	58.39	1.20	1.58E-13
GOTERM_MF_2	GO:0022892~substrate-specific transporter activity	108	9.90	1.99	2.27E-11
GOTERM_BP_2	GO:0019637~organophosphate metabolic process	47	4.31	3.10	5.75E-11
GOTERM_BP_2	GO:0044237~cellular metabolic process	613	56.19	1.17	3.41E-09
GOTERM_BP_2	GO:0055114~oxidation reduction	74	6.78	1.62	5.65E-04
GOTERM_CC_2	GO:0044425~membrane part	365	33.46	1.18	0.001424858
GOTERM_MF_2	GO:0016491~oxidoreductase activity	75	6.87	1.59	5.72E-04
GOTERM_BP_2	GO:0019222~regulation of metabolic process	335	30.71	1.19	0.001045616
GOTERM_CC_2	GO:0044464~cell part	918	84.14	1.01	0.002641755
GOTERM_CC_2	GO:0031090~organelle membrane	109	9.99	1.33	0.020285502
GOTERM_CC_2	GO:0043227~membrane-bounded organelle	636	58.30	1.07	0.026444504
GOTERM_BP_2	GO:0006066~alcohol metabolic process	49	4.49	1.52	0.04933436
GOTERM_MF_2	GO:0015238~drug transporter activity	5	0.46	5.14	0.099207977
**c) Inferred by both the human and yeast template networks**					
Category	GO Term	Number of genes	%	Fold Enrichment	Adjusted p-value
GOTERM_CC_2	GO:0044424~intracellular part	2107	86.39	1.14	1.58E-72
GOTERM_CC_2	GO:0005622~intracellular	2125	87.13	1.12	2.69E-65
GOTERM_CC_2	GO:0044446~intracellular organelle part	1135	46.54	1.41	1.27E-58
GOTERM_CC_2	GO:0044422~organelle part	1137	46.62	1.40	6.96E-58
GOTERM_MF_2	GO:0000166~nucleotide binding	698	28.62	1.61	4.71E-53
GOTERM_MF_2	GO:0001882~nucleoside binding	532	21.81	1.68	1.33E-44

GOTERM_CC_2	GO:0043229~intracellular organelle	1821	74.66	1.15	1.34E-37
GOTERM_BP_2	GO:0044237~cellular metabolic process	1534	62.89	1.18	1.51E-30
GOTERM_CC_2	GO:0043233~organelle lumen	571	23.41	1.49	6.83E-31
GOTERM_CC_2	GO:0043227~membrane-bounded organelle	1660	68.06	1.16	9.06E-31
GOTERM_CC_2	GO:0030529~ribonucleoprotein complex	220	9.02	2.00	5.31E-29
GOTERM_MF_2	GO:0003735~structural constituent of ribosome	97	3.98	2.80	4.28E-26
GOTERM_BP_2	GO:0044238~primary metabolic process	1520	62.32	1.16	1.14E-24
GOTERM_CC_2	GO:0043234~protein complex	662	27.14	1.38	3.06E-25
GOTERM_MF_2	GO:0016787~hydrolase activity	527	21.61	1.45	3.66E-24
GOTERM_CC_2	GO:0043228~non-membrane-bounded organelle	651	26.69	1.33	3.79E-20
GOTERM_BP_2	GO:0022613~ribonucleoprotein complex biogenesis	100	4.10	2.36	9.42E-19
GOTERM_BP_2	GO:0034621~cellular macromolecular complex subunit organization	145	5.95	1.93	1.07E-16
GOTERM_BP_2	GO:0045184~establishment of protein localization	252	10.33	1.57	8.99E-15
GOTERM_CC_2	GO:0044464~cell part	2215	90.82	1.02	9.44E-15
GOTERM_CC_2	GO:0031090~organelle membrane	287	11.77	1.45	1.07E-12
GOTERM_BP_2	GO:0043933~macromolecular complex subunit organization	229	9.39	1.52	9.23E-12
GOTERM_CC_2	GO:0032993~protein-DNA complex	47	1.93	2.75	1.92E-12
GOTERM_BP_2	GO:0006323~DNA packaging	55	2.26	2.39	2.19E-10
GOTERM_BP_2	GO:0009058~biosynthetic process	818	33.54	1.19	2.53E-10
GOTERM_BP_2	GO:0051649~establishment of localization in cell	255	10.46	1.44	2.42E-10
GOTERM_BP_2	GO:0009056~catabolic process	341	13.98	1.36	3.45E-10
GOTERM_BP_2	GO:0033036~macromolecule localization	300	12.30	1.37	1.54E-09
GOTERM_BP_2	GO:0006996~organelle organization	371	15.21	1.32	1.89E-09
GOTERM_CC_2	GO:0031967~organelle envelope	179	7.34	1.49	5.00E-09
GOTERM_MF_2	GO:0048037~cofactor binding	84	3.44	1.84	2.92E-08
GOTERM_BP_2	GO:0022607~cellular component assembly	248	10.17	1.38	4.12E-08
GOTERM_BP_2	GO:0051641~cellular localization	262	10.74	1.36	5.53E-08
GOTERM_MF_2	GO:0016740~transferase activity	394	16.15	1.27	8.55E-08
GOTERM_BP_2	GO:0022402~cell cycle process	175	7.18	1.46	1.79E-07
GOTERM_MF_2	GO:0008135~translation factor activity, nucleic acid binding	48	1.97	2.18	1.77E-07
GOTERM_BP_2	GO:0007049~cell cycle	223	9.14	1.36	1.42E-06
GOTERM_BP_2	GO:0006413~translational initiation	27	1.11	2.69	1.64E-06
GOTERM_MF_2	GO:0016874~ligase activity	123	5.04	1.50	4.43E-06
GOTERM_BP_2	GO:0070271~protein complex biogenesis	151	6.19	1.41	1.38E-05
GOTERM_BP_2	GO:0043170~macromolecule metabolic process	1201	49.24	1.09	1.37E-05
GOTERM_BP_2	GO:0006807~nitrogen compound metabolic process	843	34.56	1.12	3.32E-05
GOTERM_MF_2	GO:0016491~oxidoreductase activity	158	6.48	1.38	3.93E-05
GOTERM_MF_2	GO:0043021~ribonucleoprotein binding	20	0.82	2.72	6.86E-05
GOTERM_BP_2	GO:0055114~oxidation reduction	153	6.27	1.35	1.50E-04
GOTERM_BP_2	GO:0016192~vesicle-mediated transport	159	6.52	1.34	1.79E-04
GOTERM_BP_2	GO:0051301~cell division	94	3.85	1.46	3.61E-04
GOTERM_BP_2	GO:0006903~vesicle targeting	15	0.62	2.92	4.41E-04
GOTERM_BP_2	GO:0006066~alcohol metabolic process	111	4.55	1.39	6.66E-04

GOTERM_CC_2	GO:0008287~protein serine/threonine phosphatase complex	20	0.82	2.31	6.50E-04
GOTERM_MF_2	GO:0003676~nucleic acid binding	669	27.43	1.11	0.001907853
GOTERM_CC_2	GO:0012505~endomembrane system	175	7.18	1.26	0.00101062
GOTERM_BP_2	GO:0042440~pigment metabolic process	21	0.86	2.14	0.003248841
GOTERM_MF_2	GO:0016853~isomerase activity	41	1.68	1.66	0.003459648
GOTERM_BP_2	GO:0051234~establishment of localization	505	20.71	1.12	0.004572958
GOTERM_BP_2	GO:0006810~transport	499	20.46	1.12	0.004623776
GOTERM_MF_2	GO:0022892~substrate-specific transporter activity	163	6.68	1.23	0.009146655
GOTERM_MF_2	GO:0005515~protein binding	1526	62.57	1.04	0.00887686
GOTERM_BP_2	GO:0051656~establishment of organelle localization	27	1.11	1.75	0.016659579
GOTERM_BP_2	GO:0019748~secondary metabolic process	25	1.03	1.79	0.016976158
GOTERM_BP_2	GO:0051716~cellular response to stimulus	197	8.08	1.19	0.021358564
GOTERM_BP_2	GO:0016044~membrane organization	98	4.02	1.27	0.035774267
GOTERM_BP_2	GO:0055085~transmembrane transport	107	4.39	1.25	0.034843383
GOTERM_MF_2	GO:0016829~lyase activity	36	1.48	1.52	0.036316613
GOTERM_BP_2	GO:0019637~organophosphate metabolic process	52	2.13	1.39	0.047461729
GOTERM_MF_2	GO:0031202~RNA splicing factor activity, transesterification mechanism	12	0.49	2.11	0.069898287
GOTERM_CC_2	GO:0031982~vesicle	138	5.66	1.18	0.04545816
GOTERM_MF_2	GO:0019842~vitamin binding	32	1.31	1.47	0.085710514
GOTERM_CC_2	GO:0000267~cell fraction	207	8.49	1.14	0.053240678
GOTERM_MF_2	GO:0003711~transcription elongation regulator activity	11	0.45	2.11	0.088646948

Table S4. Significantly enriched GO terms in *A. fumigatus* genes of the *H. sapiens*-*A. fumigatus* network in interactions inferred a) only by the human template network b) only by the yeast template network c) by both human and yeast template network. Only GO groups with significant enrichment ($p < 0.1$) after multiple testing correction (Hochberg and Benjamini, 1990) were reported. For visualization of the gene sets see figure 2.

**a) Inferred only by the human template network**					
Category	GO Term	Number of genes	%	Fold Enrichment	Adjusted p-value
GOTERM_BP_2	GO:0055114~oxidation reduction	153	10.06	1.36	1.55E-05
GOTERM_MF_2	GO:0046906~tetrapyrrole binding	40	2.63	1.76	2.31E-04
GOTERM_MF_2	GO:0003676~nucleic acid binding	227	14.92	1.17	0.008280498
GOTERM_MF_2	GO:0016491~oxidoreductase activity	168	11.05	1.21	0.007738299
GOTERM_MF_2	GO:0043167~ion binding	225	14.79	1.15	0.015869296
GOTERM_BP_2	GO:0051716~cellular response to stimulus	41	2.70	1.46	0.07201813
GOTERM_MF_2	GO:0005515~protein binding	123	8.09	1.22	0.019686093
**b) Inferred only by the yeast template network**					
Category	GO Term	Number of genes	%	Fold Enrichment	Adjusted p-value
GOTERM_MF_2	GO:0016740~transferase activity	177	20.34	1.62	1.56E-13
GOTERM_MF_2	GO:0003700~transcription factor activity	23	2.64	3.34	1.35E-07
GOTERM_CC_2	GO:0016020~membrane	147	16.90	1.35	6.54E-06
GOTERM_CC_2	GO:0044425~membrane part	108	12.41	1.29	0.004870872
GOTERM_MF_2	GO:0015238~drug transporter activity	6	0.69	3.86	0.085658202
**c) Inferred by both the human and yeast template networks**					
Category	GO Term	Number of genes	%	Fold Enrichment	Adjusted p-value
GOTERM_MF_2	GO:0003735~structural constituent of ribosome	80	6.75	2.20	5.23E-17
GOTERM_BP_2	GO:0044237~cellular metabolic process	492	41.52	1.19	6.14E-11
GOTERM_BP_2	GO:0009058~biosynthetic process	297	25.06	1.31	9.58E-11
GOTERM_CC_2	GO:0030529~ribonucleoprotein complex	111	9.37	1.53	2.29E-08
GOTERM_BP_2	GO:0044238~primary metabolic process	497	41.94	1.14	1.43E-07
GOTERM_BP_2	GO:0043170~macromolecule metabolic process	358	30.21	1.20	2.16E-07
GOTERM_CC_2	GO:0044424~intracellular part	440	37.13	1.11	4.20E-06
GOTERM_CC_2	GO:0043228~non-membrane-bounded organelle	126	10.63	1.36	8.85E-06
GOTERM_CC_2	GO:0005622~intracellular	474	40.00	1.06	0.00128402
GOTERM_CC_2	GO:0043234~protein complex	118	9.96	1.27	0.001109787
GOTERM_BP_2	GO:0009056~catabolic process	82	6.92	1.35	0.005318356
GOTERM_BP_2	GO:0019637~organophosphate metabolic process	24	2.03	1.81	0.007708921

Table S5. Significantly enriched UniProt tissue terms in refined *H. sapiens* interactors with *A. fumigatus* as pathogen. The tissue enrichment of filtered networks was performed using inferred predictions as background. Only tissue classes with significant enrichment (p < 0.1) after multiple testing correction (Hochberg and Benjamini, 1990) were reported.

Term	Number of genes	%	Fold Enrichment	Adjusted p-value
**Platelet**	86	14.75	3.38	1.25E-21
**Fetal brain cortex**	39	6.69	3.55	1.45E-09
**B-cell lymphoma**	26	4.46	4.32	6.99E-08
**Cajal-Retzius cell**	34	5.83	3.38	7.37E-08
**T-cell**	38	6.52	2.72	2.49E-06
**Lung**	137	23.50	1.42	3.02E-04
**Epithelium**	159	27.27	1.37	3.92E-04
**Brain**	316	54.20	1.16	0.004334356
**Hepatocyte**	10	1.72	4.65	0.00632279
**Blood**	41	7.03	1.74	0.017833248
**Placenta**	157	26.93	1.25	0.030386414
**Hippocampus**	28	4.80	1.86	0.049514651
**Skin**	95	16.30	1.33	0.049439276
**B-cell**	22	3.77	2.00	0.058392955
**Colon adenocarcinoma**	11	1.89	2.84	0.083680992
**Colon carcinoma**	17	2.92	2.14	0.093551347
**Peripheral blood lymphocyte**	6	1.03	4.85	0.098941964
**Leukemic T-cell**	4	0.69	9.30	0.099834234

Table S6. Significantly enriched UniProt tissue terms in refined *H. sapiens* interactors with *C. albicans* as pathogen. The tissue enrichment of filtered networks was performed using inferred predictions as background. Only tissue classes with significant enrichment ($p < 0.1$) after multiple testing correction (Hochberg and Benjamini, 1990) were reported.

Term	Number of genes	%	Fold Enrichment	Adjusted p-value
**Platelet**	109	13.42	3.04	1.89E-24
**Fetal brain cortex**	56	6.90	3.68	1.14E-15
**Cajal-Retzius cell**	46	5.67	3.25	1.45E-10
**T-cell**	52	6.40	2.60	2.36E-08
**B-cell lymphoma**	31	3.82	3.61	4.53E-08
**Placenta**	231	28.45	1.32	4.86E-05
**Lung**	184	22.66	1.38	6.80E-05
**Epithelium**	216	26.60	1.31	2.69E-04
**Blood**	54	6.65	1.66	0.008996013
**Brain**	427	52.59	1.13	0.009583768
**Hepatocyte**	11	1.35	3.71	0.013958474
**Colon adenocarcinoma**	15	1.85	2.82	0.016896193
**Colon carcinoma**	24	2.96	2.08	0.025422689
**Liver**	137	16.87	1.28	0.024907589
**Keratinocyte**	18	2.22	2.39	0.023558143
**Pancreas**	67	8.25	1.46	0.026314904
**B-cell**	28	3.45	1.83	0.045657417
**Leukemic T-cell**	5	0.62	7.31	0.050753271
**Skin**	127	15.64	1.26	0.051148639
**Lymph**	57	7.02	1.45	0.060305289
**Fibroblast**	17	2.09	2.19	0.058832854
**Plasma**	23	2.83	1.90	0.058703507
**Pituitary**	18	2.22	2.11	0.058294966
**Urinary bladder**	21	2.59	1.92	0.074998095
**Peripheral blood lymphocyte**	7	0.86	4.00	0.073386523
**Cervix**	43	5.30	1.50	0.08498856
**Lymphoma**	9	1.11	3.04	0.086617158
**Bone**	9	1.11	2.96	0.097147898
